# Ca^2+^/Calmodulin-Dependent Protein Kinase II Regulation by RIPK3 Alleviates Necroptosis in Transverse Arch Constriction-Induced Heart Failure

**DOI:** 10.3389/fcvm.2022.847362

**Published:** 2022-04-28

**Authors:** Ji Cao, Jingjing Zhang, Jianan Qian, Xue Wang, Wei Zhang, Xiangfan Chen

**Affiliations:** ^1^School of Pharmacy, Nantong University, Nantong, China; ^2^School of Medicine, Nantong University, Nantong, China; ^3^Department of Pharmacy, Nantong First People’s Hospital, The Second Affiliated Hospital of Nantong University, Nantong, China

**Keywords:** receptor interacting protein kinase 3, necroptosis, heart failure, RIPK3, Ca^2+^/calmodulin-dependent protein kinase IIδ

## Abstract

Some studies have reported that the activation of Ca^2+^/calmodulin dependent protein kinase (CaMKII) plays a vital role in the pathogenesis of cardiovascular disease. Moreover, receptor interacting protein kinase 3 (RIPK3)-mediated necroptosis is also involved in the pathological process of various heart diseases. In the present study, we aimed to investigate the effect of RIPK3-regulated CaMKII on necroptosis in heart failure (HF) and its underlying mechanism. Wild type (WT) and RIPK3-depleted (RIPK3^–/–^) mice were treated with transverse arch constriction (TAC). After 6 weeks, echocardiography, myocardial injury, CaMKII activity, necroptosis, RIPK3 expression, mixed lineage kinase domain-like protein (MLKL) phosphorylation, and mitochondrial ultrastructure were measured. The results showed that TAC aggravated cardiac dysfunction, CaMKII activation, and necroptosis in WT mice. However, depletion of RIPK3 alleviated cardiac insufficiency, CaMKII activation, and necroptosis in TAC-treated mice. To verify the experimental results, WT mice were transfected with AAV-vector and AAV-RIPK3 shRNA, followed by TAC operation. The findings were consistent with the expected results. Collectively, our current data indicated that the activation of CaMKII, MLKL and necroptosis in HF mice were increased in a RIPK3-dependent manner, providing valuable insights into the pathogenesis and treatment strategy of HF.

## Introduction

As a complex clinical syndrome, heart failure (HF) is caused by ventricular dysfunction or structural damage, leading to insufficient cardiac output, as well as the inability to meet the body’s metabolic needs. Despite progress in recent years in treatment and prevention, HF is still the leading cause of incidence rate and mortality of cardiovascular diseases worldwide. About 40 million people suffer from HF globally (1). HF is pathologically characterized by myocardial stiffness, arrhythmia, myocardial fibrosis, and myocardial hypertrophy. The pathogenesis of HF may be attributed to many factors, while the most common risk factors are hypertension, coronary heart disease (heart artery obstruction), diabetes, obesity, smoking, and genetics (2, 3). However, effective therapeutic targets and approaches for HF are not well established. Therefore, it is urgently necessary to develop potential control strategies.

Recent studies have found a new type of apoptosis, namely necroptosis, morphologically different from apoptosis or necrosis (4). Necroptosis is mainly characterized by cell enlargement, swelling of organelles (especially mitochondria), rupture of the plasma membrane, cell disintegration, loss of integrity, and release of pro-inflammatory contents (5, 6). Receptor interacting protein kinase 3 (RIPK3) plays a crucial role in the signaling pathway of necroptosis. RIPK3 triggers necroptosis together with receptor-interacting protein 1 (RIPK1) and mixed line kinase domain-like protein (MLKL) (7, 8). In addition, studies have found that Ca^2+^/calmodulin-dependent protein kinase (CaMKII) is a new substrate of RIPK3-mediated myocardial necroptosis caused by ischemia and oxidative stress (9). Furthermore, new evidence has confirmed that necroptosis plays a vital role in cardiovascular disease (10). CaMKII is a serine/threonine-specific phosphokinase with multiple functions, including critical proteins regulating Ca^2+^ treatment, intercellular coupling, cell death, inflammation, and mitochondrial function (11–13). CaMKII can be activated by binding to calcium bound calmodulin (Ca^2+^/CAM), autophosphorylation, oxidation, S-nitrosylation, and o-glutamate acylation (14–17). The activation of CaMKII mediates the physiological or pathological response and remodeling under cardiac stress. Studies have shown that continuous CaMKII activation plays a vital role in HF (18, 19), myocardial hypertrophy (20), arrhythmia (21), and myocardial infarction (22). In recent years, some studies have revealed the potential role of CaMKII in HF (23). Therefore, clarifying the mechanism of CaMKII may help provide new pharmacological targets for HF.

CaMKII has four subtypes: α, β, γ, and δ. CaMKIIδ is particularly important in the heart. There are three isoforms of CaMKIIδ resulting from the alternative splicing of exons 14, 15, and 16 of its pre-mRNA, CaMKIIδA, CaMKIIδ B, and CaMKIIδ C. CaMKIIδ alternative splicing is strictly regulated. Once disorder occurs, the expressions of the three variants become unbalanced, resulting in cardiomyocyte dysfunction and eventually heart disease (24).

RIPK3 is a serine/threonine kinase mainly present in the heart. It activates CaMKII through phosphorylation and/or oxidation, triggering mitochondrial permeability transition pore (MPTP) opening and myocardial necroptosis (9). Studies have shown that the expression of RIPK3 is elevated in HF patients (25). However, the clinical value of RIPK3 remains largely undetermined.

In the present study, we investigated the effect of CaMKII on necroptosis in the development of HF and its molecular mechanism. In addition, we also explored the regulatory effect of RIPK3 on CaMKII δ alternative splicing and CaMKII activity to ameliorate transverse arch constriction (TAC)-induced myocardial necroptosis in wild type (WT) and RIPK3^–/–^ mice with HF.

## Materials and Methods

### Animals

Wild type C57BL/6 mice (male, 8 weeks old) were provided by the Experimental Animal Center of Nantong University (Nantong, China). RIPK3^–/–^ mice were donated by the Institute of Molecular Medicine, Peking University (Beijing, China). The animals were housed in the Experimental Animal Center of Nantong University at 20°C with a 12-h light/dark cycle, and the mice were fed a standard laboratory diet and tap water. All animal-related procedures complied with the recommendations of the “Guidelines for the Care and Use of Laboratory Animals” (approval number: NTU-20161225) issued by the National Institutes of Health and the Animal Care and Use Steering Committee of Nantong University.

### Establishment of Heart Failure Mouse Model

Mice were randomly divided into WT mice group (WT), WT mice receiving TAC (26) treatment group (WT + TAC), RIPK3^–/–^ mice group (RIPK3^–/–^), and RIPK3^–/–^ mice receiving TAC treatment group (RIPK3^–/–^ + TAC). After 1 week of adaptive feeding, the mice in the model group underwent TAC after overnight food deprivation. Briefly, the mice were intraperitoneally injected with chloral hydrate and depilated after anesthesia. First, the neck skin was cut along the midline, followed by intubation. Next, the sternum was cut to expose the thymus, the thymus was separated, and then the aortic arch was identified and narrowed with a 28-g needle. The mice in the control group received sham surgery. All groups of mice were given continuous feeding for 6 weeks.

### Tail Vein Injection of Adeno-Associated Virus

To evaluate the role of RIPK3 in HF, male C57BL/6 mice (8 weeks old) were transfected with Adeno-Associated Virus (AAV)-vector and AAV-RIPK3 shRNAs through the tail vein. The injection dose was 1 × 10^11vg per mouse once, and then TAC operation was performed to establish a mouse model of HF. The operation method was the same as those mentioned above. In addition, all groups of mice were given continuous feeding for 6 weeks.

### Echocardiography

At 6 weeks after the operation, the mice were anesthetized with 1.5% isoflurane, the left chest hair was removed, and the heart geometry was measured at a probe frequency of 30 MHz from the parasternal long-axis view using a small animal color ultrasonic diagnostic device. The cardiac morphology and function were examined by two-dimensional guided M-mode echocardiography (visual sonic VEVO 2100, Toronto, ON, Canada). The thickness of the interventricular septum (IVS) and left ventricle posterior wall (LVPW) was determined. Subsequently, the ejection fraction (EF) and LV fractional shortening (FS) were calculated based on the average of 10 cardiac cycles. All indexes were measured three times in a row.

### Hematoxylin and Eosin Staining

The left ventricles were fixed overnight in 4% paraformaldehyde, embedded in paraffin, and then cut into 5-μm sections. The sections were subjected to Hematoxylin and Eosin (H&E) staining and dehydrated with ethanol. Subsequently, the sections were observed and photographed under an optical microscope.

### TdT-Mediated dUTP Nick-End Labeling Staining

The left ventricles were fixed overnight in 4% paraformaldehyde, embedded in paraffin, and then cut into 5-μm sections. After staining with TdT-Mediated dUTP Nick-End Labeling (TUNEL) (Beyotime, Shanghai, China) at 37°C for 60 min, the sections were washed three times with PBS, then observed, and photographed under an optical microscope. The quantification was performed using ImageJ software.

### Sirius Red Staining

The left ventricles were fixed overnight in 4% paraformaldehyde, embedded in paraffin, and then cut into 5-μm sections. After staining with Weigert’s iron hematoxylin staining solution and then drip staining with Sirius scarlet staining solution, the sections were conventionally dehydrated to transparent, sealed with neutral gum, and photographed under a microscope.

### Masson Staining

The left ventricles were fixed overnight in 4% paraformaldehyde, embedded in paraffin, and then cut into 5-μm sections. The paraffin-embedded sections were deparaffinized in water, chromated with potassium dichromate overnight, stained with iron hematoxylin, ponceau acid fuchsin, phosphomolybdic acid, and aniline blue sequentially, then dehydrated, mounted, and examined under a microscope.

### WGA Staining

The left ventricles were fixed overnight in 4% paraformaldehyde, embedded in paraffin, and then cut into 5-μm sections. Next, the paraffin-embedded sections were deparaffinized in water. After antigen retrieval, the WGA working solution was added dropwise, followed by incubation for 30 min. Subsequently, the sections were stained for nuclear mounting and examined under a microscope.

### Determination of Blood Biochemical Indexes

Before sacrifice, whole blood was collected from the orbital vein of each mouse and centrifuged at 3,000 *g* for 20 min. Serum was kept at −80°C for other analyses. Corresponding commercial kits were used to detect the lactate dehydrogenase (LDH) activity and the contents of creatine kinase (CK), interleukin-6 (IL-6), and tumor necrosis factor-α (TNF-α) in mouse serum.

### Measurement of Superoxide Formation

Superoxide production in myocardial tissue was detected using dihydroethidium (DHE) staining under a fluorescence microscope. Briefly, myocardial tissue sections (5 μm) were prepared and then incubated (30 min, 37°C) in Krebs-HEPES buffer (mM components: NaCl 99, KCl 4.7, MgSO_4_ 1.2, KH2 PO_4_ 1.0, CaCl_2_ 1.9, NaHCO_3_ 25, glucose 11.1, Na HEPES 20; pH 7.4) containing 2 μM DHE in a dark room. The slides were examined with a Nikon TE2000 inverted microscope (Nikon, Tokyo, Japan) at excitation and emission wavelengths of 480 and 610 nm, respectively.

The thiobarbituric acid method (Beyotime) was used to detect the level of malondialdehyde (MDA) in the myocardium. The total antioxidant capacity (T-AOC) of the myocardium was measured by the T-AOC detection kit using the plasma iron reduction capacity method (Beyotime). The activities of total superoxide dismutase (SOD) in the myocardium were determined using the WST-1 (2-(4-iodophenyl)-3-(4-nitrophenyl)-5-(2,4-disulfophenyl)-)-2 Htetrazole) method (Beyotime).

### Ultrastructural Observation of Myocardium

The fresh myocardia were cut into three pieces (1 mm) and fixed with 4% glutaraldehyde and 1% osmium acid. The samples were dehydrated with acetone, embedded in Epon812, stained with toluidine blue, cut into 70-nm sections, and stained with uranyl acetate and lead citrate. A transmission electron microscope (JEM-1230) was used to examine the ultrastructure of myocardial tissue. A visible image consisting of 15 randomly selected areas per slice was taken to determine the structure and number of mitochondria. The volume of mitochondria was calculated, and the number of mitochondria was counted.

### Quantitative Real-Time Polymerase Chain Reaction

Total RNA was extracted from myocardial tissue using Trizol reagent (Takara, Kyoto, Japan), and then the purified RNA was reversely transcribed into cDNA using PrimeScript™ RT Master Mix Kit (Takara). SYBR Green Fast qPCR mix (Takara) and ABI 7500 real-time PCR system (ABI, Carlsbad, CA, United States) were used to perform Quantitative Real-Time Polymerase Chain Reaction (qRT-PCR). 18S was used as a housekeeping gene. Each experiment was performed in triplicate. The relative expressions of target genes were calculated using the 2^–ΔΔ*Ct*^ method.

### Western Blotting Analysis

The proteins extracted from the myocardial tissue were subjected to SDS-PAGE and transferred onto the PVDF membranes (Millipore, Billerica, MA, United States). After blocking with TBST buffer (Tris-HCl 10 mmol⋅L-1, NaCl 120 mmol⋅L-1, Tween-20 0.1%; pH 7.4) containing 5% skimmed milk (v/v) at room temperature for 2 h, the membranes were incubated with primary antibodies at 4°C overnight, including anti-ANP (1:1,000, Abcam, Cambridge, United Kingdom), anti-BNP (1:1,000, Abcam, Cambridge, United Kingdom), anti-ox-CaMKII (1: 1,000, Millipore, Kenilworth, NJ, United States), anti-CaMKII (1: 1,000, Abcam, Cambridge, United Kingdom), anti-caspase 3, anti-cleaved caspase 3, anti-MLKL, anti-p-MLKL, and anti-RIPK1 (1: 1,000, Cell Signaling Technology, Danvers, MA, United States); anti-p-CaMKII (1: 1,000, Thermo Fisher Scientific, Rockford, IL, United States), anti-GAPDH (1: 5,000, Sigma-Aldrich, St. Louis, MO, United States), and anti-β-tubulin. Subsequently, the blots were incubated with horseradish peroxidase (HRP-)-conjugated secondary antibody at room temperature for 1.5 h. The immunoreactive bands were visualized by enhanced chemiluminescence (ECL) (Thermo Fisher Scientific, Rockford, IL, United States). GAPDH or β-tubulin was used as the loading control.

### Statistical Analysis

All data were expressed as mean ± standard error (SEM). Comparisons between two groups were conducted by the unpaired Student’s *t*-test. One-way analysis of variance analysis (ANOVA) and Student-Newman-Keuls (SNK) test statistics were used for comparisons among multiple groups. *P* < 0.05 was considered statistically significant.

## Results

### Cardiac Dysfunction, CaMKIIδ Alternative Splicing Disorder, and Enhancement of Apoptosis in Heart Failure Mice

**FIGURE 1:**
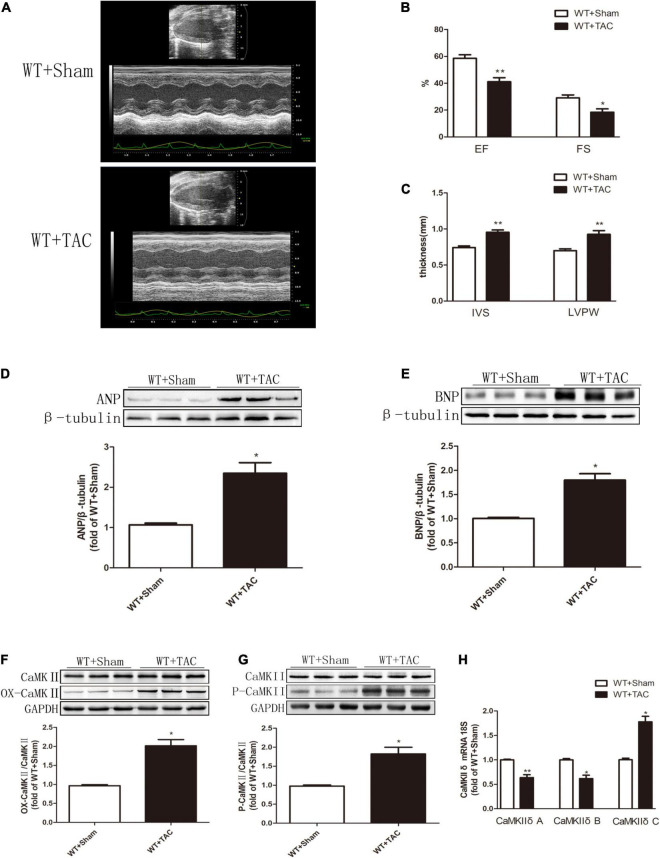
Cardiac dysfunction, increased expressions of ANP and BNP, CaMKIIδ alternative splicing disorder, CaMKII oxidation, and phosphorylation levels are increased in HF mice. Male 8-week-old C57BL/6 mice were treated with TAC using a 28-g needle. The control group received sham surgery. All groups of mice were given continuous feeding for 6 weeks. Hearts were then obtained for correlative testing. **(A)** Echocardiography. **(B)** EF and FS. **(C)** IVS and LVPW. **(D)** The expression of ANP at the protein level in myocardial tissue was detected by Western blotting analysis, and β-tubulin was used as a loading control; **(E)** The expression of BNP at the protein level in myocardial tissue was detected by Western blotting analysis, and β-tubulin was used as a loading control. **(F)** Western blotting analysis was used to detect the expression of OX-CaMKII protein in myocardial tissue, and GAPDH was used as loading control; **(G)** Western blotting was used to detect the expression of P-CaMKII protein in myocardial tissue, and GAPDH was used as a loading control. **(H)** Detection of myocardial CaMKIIδ A, CaMKIIδ B, and CaMKIIδ C at the mRNA level by qRT-PCR, and 18S was used as the housekeeping gene. Compared with the WT + sham group, **P < 0.01 and *P < 0.05. *n* = 6.

**FIGURE 2:**
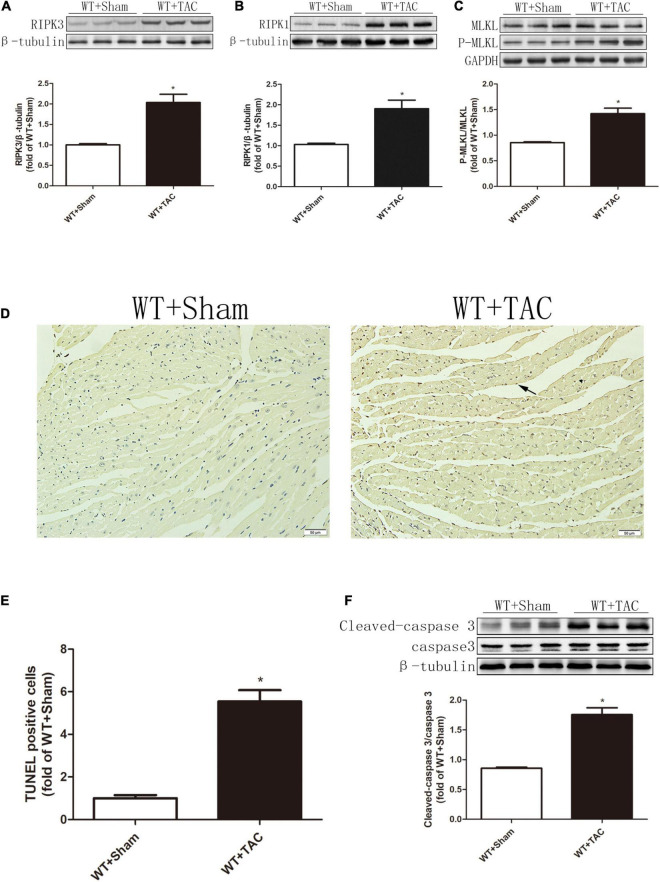
The expressions of RIPK3 and RIPK1, as well as the levels of MLKL phosphorylation and apoptosis, are increased in HF mice. Male 8-week-old C57BL/6 mice were treated with TAC using a 28-g needle. The control group received sham surgery. All groups of mice were given continuous feeding for 6 weeks. Hearts were then obtained for correlative testing. **(A)** The expression of RIPK3 at the protein level in myocardial tissue was detected by Western blotting analysis, and β-tubulin was used as a loading control. **(B)** The expression of RIPK1 at the protein level in myocardial tissue was detected by Western blotting analysis, and β-tubulin was used as a loading control. **(C)** The levels of MLKL and P-MLKL proteins in myocardial tissue were detected by Western blotting analysis, and GAPDH was used as a loading control. **(D)** The level of apoptosis in myocardial tissue was measured by TUNEL staining, bar = 50 μm; **(E)** The statistical chart of TUNEL-positive cells. **(F)** The levels of cleaved caspase 3 and caspase 3 in myocardial tissue were detected by Western blotting analysis, and β-tubulin was used as a loading control. Compared with WT + sham group, *P < 0.05. *n* = 6.

Echocardiography showed that compared with the control group, HF mice had diastolic and systolic dysfunctions, the levels of EF, FS, and E/A were decreased (Figures 1A–C), and the expressions of hypertrophy and HF indexes ANP and BNP were significantly increased (Figures 1D,E), indicating that an HF model was successfully established in this study. CaMKIIδ alternative splicing disorder easily promotes cardiomyocyte dysfunction and ultimately leads to heart disease (27). Since there is no specific antibody for CaMKIIδ splice variants, qRT-PCR was used to detect the expressions of CaMKIIδ A, CaMKIIδ B, and CaMKIIδ C at the mRNA level. In HF mice, the expressions of CaMKIIδ A and CaMKIIδ B were significantly decreased, while the expression of CaMKIIδ C was significantly increased (Figure 1H), indicating CaMKIIδ alternative splicing disorder in HF mice. In addition, the oxidation and phosphorylation of CaMKII were also increased in the HF group (Figures 1F,G). Several studies have shown that RIPK3 is involved in necroptosis. Our study confirmed that RIPK3 was significantly increased in the heart of HF mice (Figure 2A). Meanwhile, the expression of RIPK1 downstream of RIPK3 and the phosphorylation of MLKL were also up-regulated in the myocardium of HF mice (Figures 2B,C). Necroptosis is a particular form of cell death. TUNEL staining and the expression of cleaved-caspase 3 (Figures 2D–F) showed significantly more apoptotic cardiomyocytes in HF mice compared with the control group. All these data showed that cardiac dysfunction, CaMKII δ activity, alternative splicing disorder, and apoptosis were enhanced in HF mice.

### RIPK3 Deficiency Can Reduce Cardiac Dysfunction, Myocardial Injury, Myocardial Fibrosis, and Inflammatory Response in Heart Failure

**FIGURE 3:**
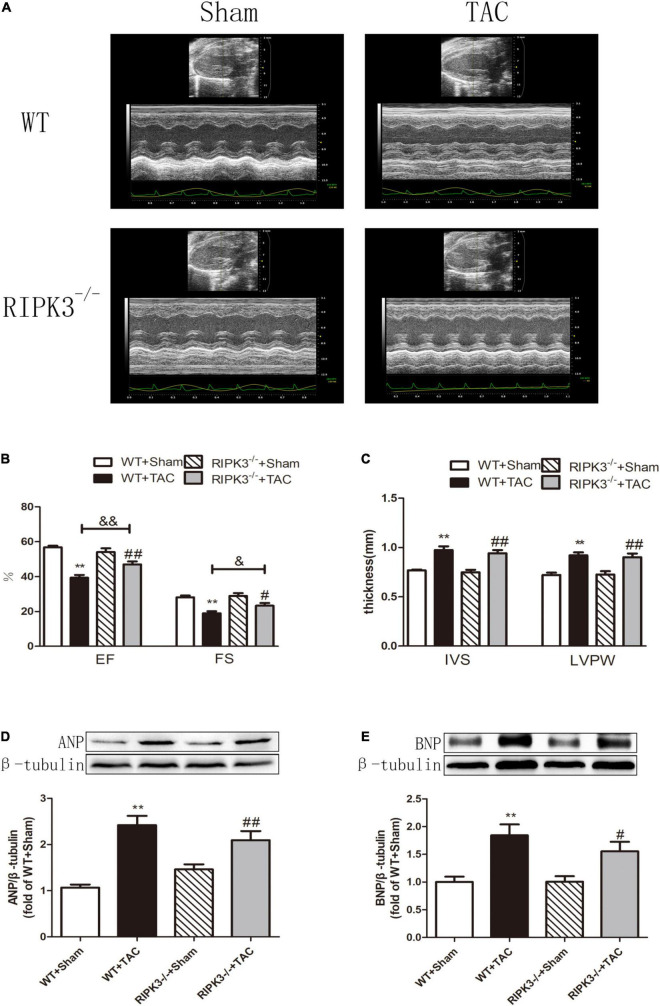
Depletion of RIPK3 can alleviate cardiac insufficiency in mice with HF and has no significant improvement in the increased expressions of ANP and BNP. Male 8-week-old WT and RIPK3^–/–^ mice were treated with TAC using a 28-g needle. The control group received sham surgery. All groups of mice were given continuous feeding for 6 weeks. Hearts were then obtained for correlative testing. **(A)** Echocardiography. **(B)** EF and FS. **(C)** IVS and LVPW. **(D)** The expression of ANP at the protein level in myocardial tissue was detected by Western blotting analysis, and β-tubulin was used as a loading control; **(E)** The expression of BNP at the protein level in myocardial tissue was detected by Western blotting analysis, and β-tubulin was used as a loading control. Compared with WT + sham group, **P < 0.01; compared with RIPK3^–/–^ + sham group, ^##^*P* < 0.01 and ^#^*P* < 0.05; compared with WT + TAC group, ^&⁣&^*P* < 0.01 and ^&^*P* < 0.05. *n* = 6.

**FIGURE 4:**
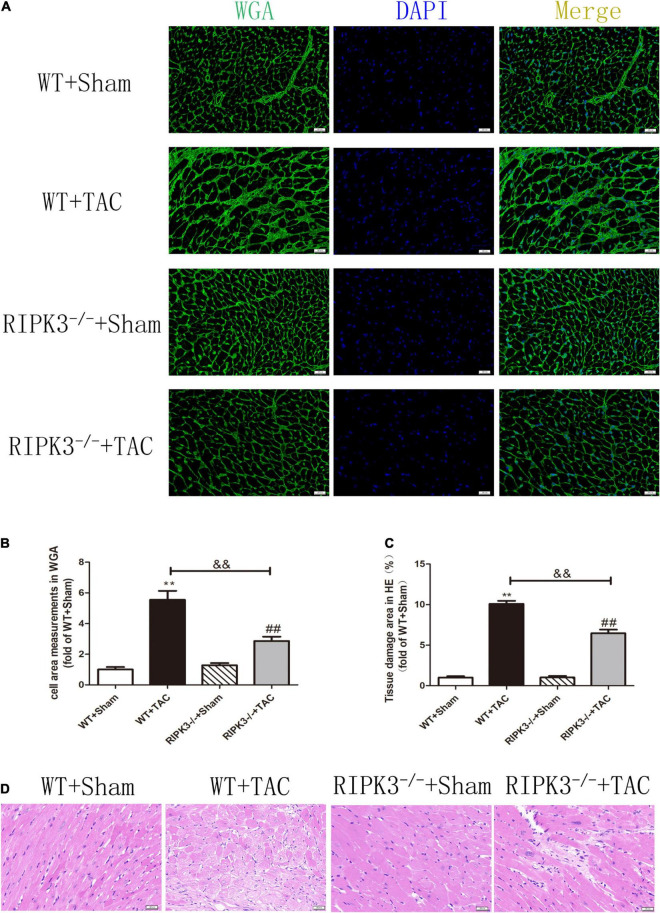
Depletion of RIPK3 can inhibit the increase of cardiomyocyte cross-sectional area in mice with HF and improve myocardial injury. Male 8-week-old WT and RIPK3^–/–^ mice were treated with TAC using a 28-g needle. The control group received sham surgery. All groups of mice were given continuous feeding for 6 weeks. Hearts were then obtained for correlative testing. **(A)** The cross-sectional area of mouse cardiomyocytes was observed by WGA staining, bar = 50 μm. **(B)** Cell area measurements in WGA. **(C)** Tissue damage area measurements in H&E. **(D)** H&E staining was used to detect the degree of myocardial injury, bar = 50 μm. Compared with WT + sham group, **P < 0.01; compared with RIPK3^–/–^ + sham group, ^##^*P* < 0.01; compared with WT + TAC group, ^&&^*P* < 0.01. *n* = 6.

**FIGURE 5:**
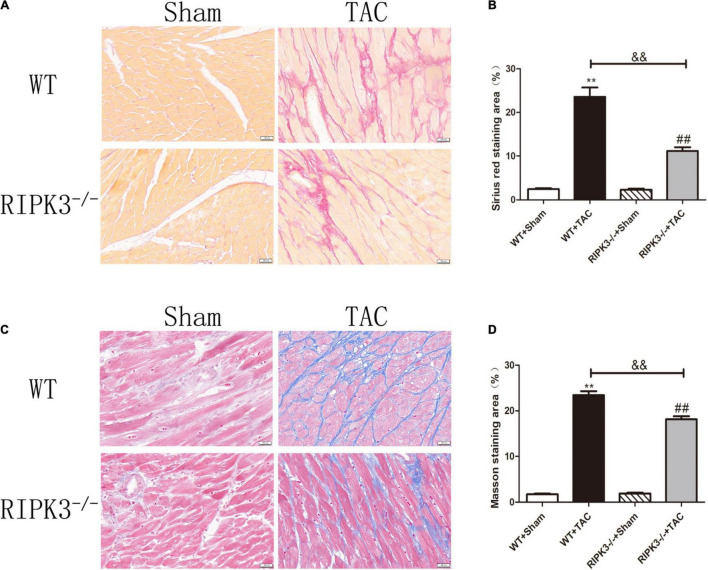
Depletion of RIPK3 can improve the degree of collagen deposition and myocardial fibrosis in HF mice. Male 8-week-old WT and RIPK3^–/–^ mice were treated with TAC using a 28-g needle. The control group received sham surgery. All groups of mice were given continuous feeding for 6 weeks. Hearts were then obtained for correlative testing. **(A)** Sirius red staining, bar = 50 μm. **(B)** Sirius red staining area measurements. **(C)** Masson staining, bar = 50 μm. **(D)** Masson staining area measurements. Compared with WT + sham group, **P < 0.01; compared with RIPK3^–/–^ + sham group, ^##^*P* < 0.01; compared with WT + TAC group, ^&&^*P* < 0.01. *n* = 6.

**FIGURE 6:**
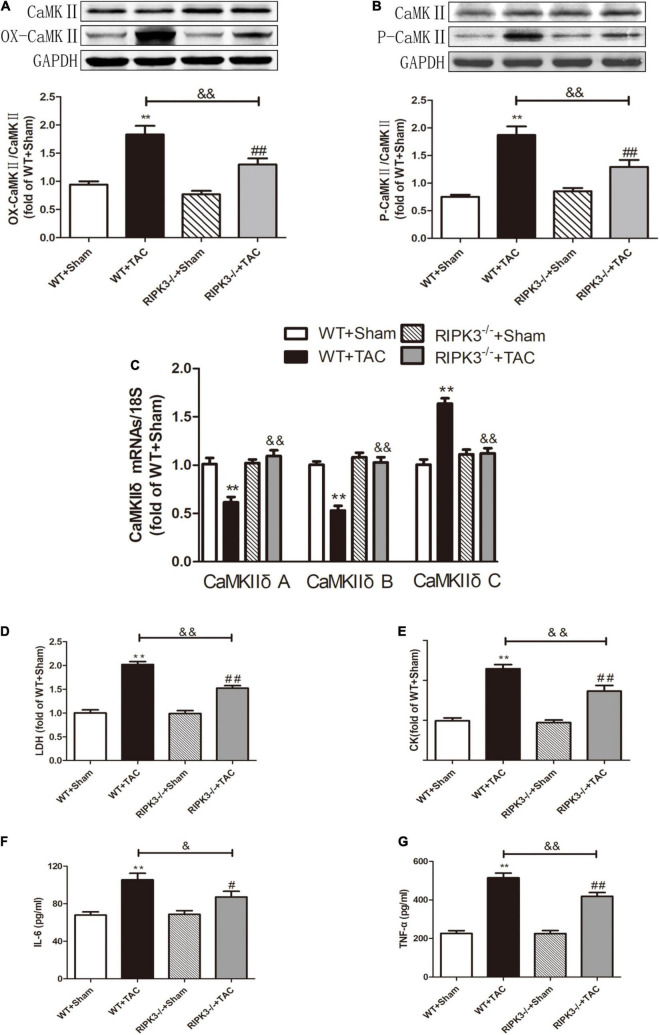
Depletion of RIPK3 can alleviate the elevated levels of CaMKII oxidation and phosphorylation, improve the CaMKIIδ alternative splicing disorder in HF mice and the degree of myocardial injury and inflammatory response. Male 8-week-old WT and RIPK3^–/–^ mice were treated with TAC using a 28-g needle. The control group received sham surgery. All groups of mice were given continuous feeding for 6 weeks. Hearts were then obtained for correlative testing. **(A)** The expression of OX-CaMKII at the protein level in myocardial tissue was detected by Western blotting analysis, and GAPDH was used as a loading control. **(B)** The expression of P-CaMKII at the protein level in myocardial tissue was detected by Western blotting analysis, and GAPDH was used as a loading control. **(C)** Detection of myocardial CaMKIIδ A, CaMKIIδ B, and CaMKIIδ C at the mRNA level by qRT-PCR, and 18S was used as the housekeeping gene. **(D)** The levels of serum LDH, **(E)** CK, **(F)** IL-6, and **(G)** TNF-α. Compared with WT + sham group, **P < 0.01; compared with RIPK3^–/–^ + sham group, ^##^*P* < 0.01 and ^#^*P* < 0.05; compared with WT + TAC group, ^&⁣&^*P* < 0.01 and ^&^*P* < 0.05. *n* = 6.

We used RIPK3^–/–^ mice to verify the role of RIPK3 in the development of HF. Echocardiography showed that depletion of RIPK3 significantly increased EF and FS in HF mice compared with the control group (Figures 3A–C), indicating that systolic and diastolic functions were improved. By detecting the expressions of ANP and BNP at the protein level, we found that depletion of RIPK3 had no significant effect on hypertrophy and expressions of HF genes in HF mice (Figures 3D,E). The cross-sectional area of mouse cardiomyocytes was determined by WGA staining, indicating that cardiomyocytes of WT and RIPK3^–/–^ mice were significantly enlarged after TAC operation, and depletion of RIPK3 could significantly reduce the size of cardiomyocytes (Figures 4A,B). H&E staining showed that depletion of RIPK3 could reduce the distortion and disorder of cardiomyocytes in HF mice (Figures 4C,D). To evaluate the degree of myocardial fibrosis in mice, we applied Sirius red staining (Figures 5A,B) and Masson staining (Figures 5C,D). The results showed that depletion of RIPK3 significantly improved the degree of collagen deposition and myocardial fibrosis. After TAC operation, the serum LDH and CK levels of RIPK3^–/–^ mice were significantly lower compared with WT mice (Figures 6D,E). Depletion of RIPK3 improved the degree of myocardial injury in HF mice. Moreover, we found that the inflammatory level was significantly decreased in the myocardial tissue in the RIPK3^–/–^ + TAC group (Figures 6F,G), indicating that the myocardial injury was improved to a certain extent. These data suggested that although depletion of RIPK3 could not significantly inhibit the occurrence and development of HF, it could significantly ameliorate the central dysfunction, myocardial injury, myocardial fibrosis, and inflammatory response in HF.

### Loss of RIPK3 Can Reduce RIPK1 Expression, MLKL Phosphorylation, Necroptosis, CaMKII Activity, and CaMKIIδ Alternative Splicing Disorder in Heart Failure Mice and Improve Oxidative Stress and Myocardial Mitochondrial Ultrastructure

**FIGURE 7:**
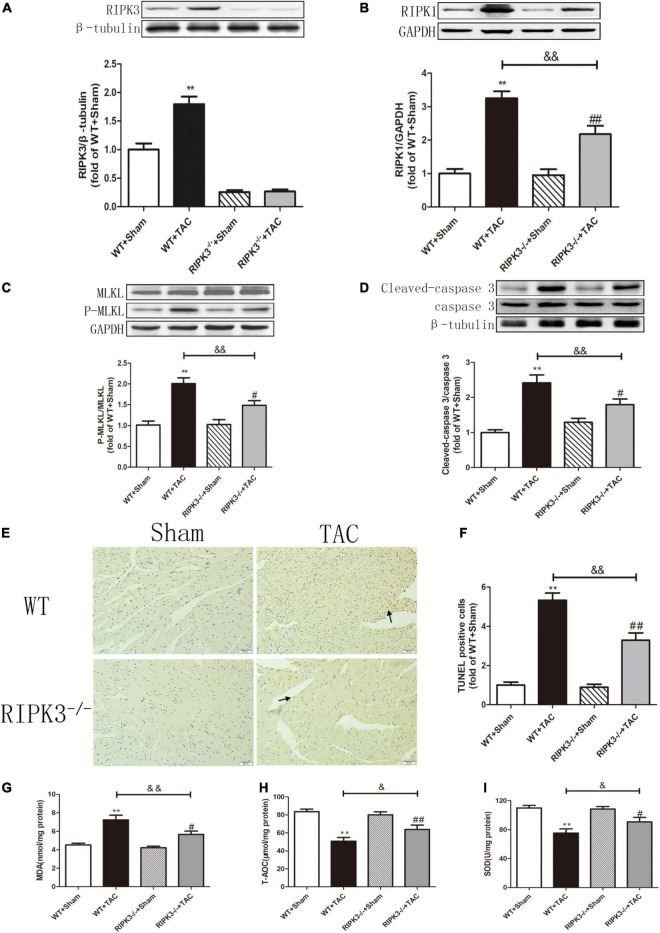
Depletion of RIPK3 can reduce the expressions of RIPK3 and RIPK1, as well as the MLKL phosphorylation level and cardiomyocyte apoptosis in HF mice and improve antioxidant capacity. Male 8-week-old WT and RIPK3^–/–^ mice were treated with TAC using a 28-g needle. The control group received sham surgery. All groups of mice were given continuous feeding for 6 weeks. Hearts were then obtained for correlative testing. **(A)** The expression of RIPK3 at the protein level in myocardial tissue was detected by Western blotting analysis, and β-tubulin was used as a loading control. **(B)** Western blotting analysis was used to detect the expression of RIPK1 at the protein level in myocardial tissue, and GAPDH was used as a loading control. **(C)** Western blotting analysis was used to detect the expressions of MLKL and P-MLKL at the protein level in myocardial tissue, and GAPDH was used as a loading control. **(D)** The expressions of cleaved caspase 3 and caspase 3 at the protein level in myocardial tissue were detected by Western blotting analysis, and β-tubulin was used as a loading control. **(E)** TUNEL staining was used to determine the level of apoptosis in myocardial tissue, bar = 50 μm. **(F)** The statistical chart of TUNEL-positive cells. **(G)** The levels of serum MDA, **(H)** T-AOC, and **(I)** SOD. Compared with WT + sham group, **P < 0.01; compared with RIPK3^–/–^ + sham group, ^##^*P* < 0.01 and ^#^*P* < 0.05; compared with WT + TAC group, ^&⁣&^*P* < 0.01 and ^&^*P* < 0.05. *n* = 6.

**FIGURE 8:**
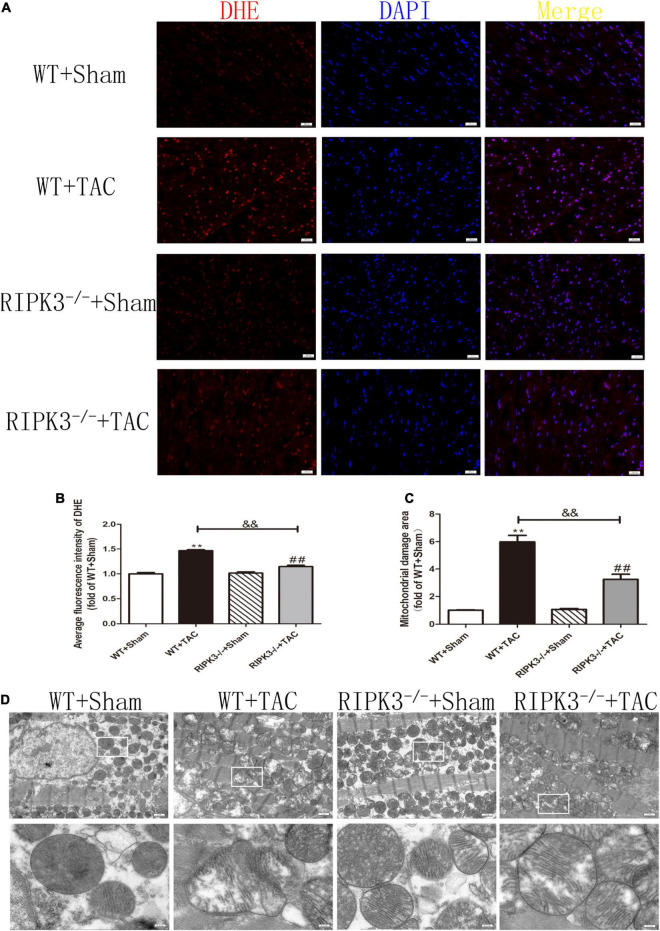
Depletion of RIPK3 can reduce ROS accumulation in myocardial tissue of HF mice and improve antioxidant capacity and mitochondrial structural abnormalities. Male 8-week-old WT and RIPK3^–/–^ mice were treated with TAC using a 28-g needle. The control group received sham surgery. All groups of mice were given continuous feeding for 6 weeks. Hearts were then obtained for correlative testing. **(A)** DHE staining, bar = 50 μm. **(B)** Average fluorescence intensity measurements of DHE. **(C)** Mitochondrial damage area. **(D)** The ultrastructure of LV mitochondria was observed by transmission electron microscope, bar = 2 μm. Lower picture bar = 0.5 μm. Compared with WT + sham group, **P < 0.01; compared with RIPK3^–/–^ + sham group, ^##^*P* < 0.01; compared with WT + TAC group, ^&&^*P* < 0.01. *n* = 6.

Our results showed that depletion of RIPK3 could reduce the oxidation and phosphorylation of CaMKII in the myocardium of HF mice (Figures 6A,B). The expression of CaMKIIδ was detected by qRT-PCR. We found that depletion of RIPK3 improved CaMKIIδ alternative splicing disorder to some extent (Figure 6C), which alleviated cardiomyocyte disorder. In addition to RIPK3, studies have shown that RIPK1 is also involved in necroptosis, and the phosphorylation of MLKL is an essential effector molecule of necroptosis (28). In the present study, the expression of RIPK3 was significantly increased in the myocardium of HF mice (Figure 7A), while the RIPK1 expression and MLKL phosphorylation were also increased. However, the RIPK1 expression and MLKL phosphorylation were reduced in RIPK3^–/–^ HF mice (Figures 7B,C). TUNEL staining and the expression of cleaved caspase 3 (Figures 7D–F) showed that apoptosis was significantly improved in RIPK3^–/–^ HF mice. In addition, oxidative stress plays an essential role in the occurrence and development of heart disease. Reactive oxygen species (ROS) accumulation is the main factor in myocardial necrosis, which may reduce the survival of myocardial cells and aggravate myocardial damage. We selected MDA, T-AOC, and T-SOD kits to evaluate the AOC of mouse myocardial tissue, and the results showed that the myocardial tissue’s ability to scavenge oxygen free radicals was enhanced in RIPK3^–/–^ mice, and the accumulation of ROS was decreased (Figures 7G–I). We used DHE staining to evaluate the level of ROS in tissues. We found that at 6 weeks after the TAC surgery, the red fluorescence intensity of myocardial tissue in WT mice was increased, while the red fluorescence intensity of myocardial tissue in RIPK3^–/–^ mice was weaker compared with WT mice, indicating that depletion of RIPK3 reduced the accumulation of ROS in myocardial tissue (Figures 8A,B). The ultrastructure of myocardial mitochondria in the left ventricle of mice with myocardial hypertrophy was observed by a transmission electron microscope. However, in contrast to WT and RIPK3^–/–^ mice without TAC, mitochondria in the RIPK3^–/–^ + TAC group were relatively intact and began to exhibit cristae fragmentation, which was much improved compared with the WT + TAC group (Figures 8C,D).

### AAV-RIPK3 shRNA Interferes With the Expression of RIPK3, Reverses the Dysfunction of Myocardial Hypertrophy, Myocardial Fibrosis, and Inflammation, and Reduces Myocardial Damage

**FIGURE 9:**
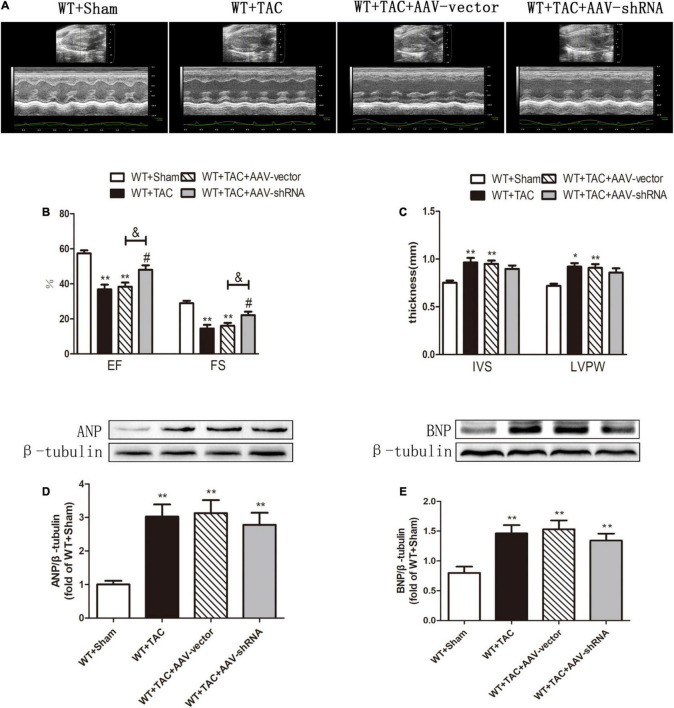
Interference with RIPK3 can alleviate cardiac insufficiency in HF mice and has no significant improvement in the up-regulation of ANP and BNP. Male 8-week-old WT mice were pretreated with AAV-vector and AAV-shRNA through the tail vein, and TAC was performed using a 28-g needle. The control group received sham surgery. All groups of mice were given continuous feeding for 6 weeks. Hearts were then obtained for correlative testing. **(A)** Echocardiography. **(B)** EF and FS. **(C)** IVS and LVPW. **(D)** The expression of ANP at the protein level in myocardial tissue was detected by Western blotting analysis, and β-tubulin was used as a loading control. **(E)** The expression of BNP at the protein level in myocardial tissue was detected by Western blotting analysis, and β-tubulin was used as a loading control. Compared with WT + sham group, **P < 0.01 and *P < 0.05; compared with WT + TAC group, ^#^*P* < 0.05; compared with WT + AAV-vector group, ^&^*P* < 0.05. *n* = 6.

**FIGURE 10:**
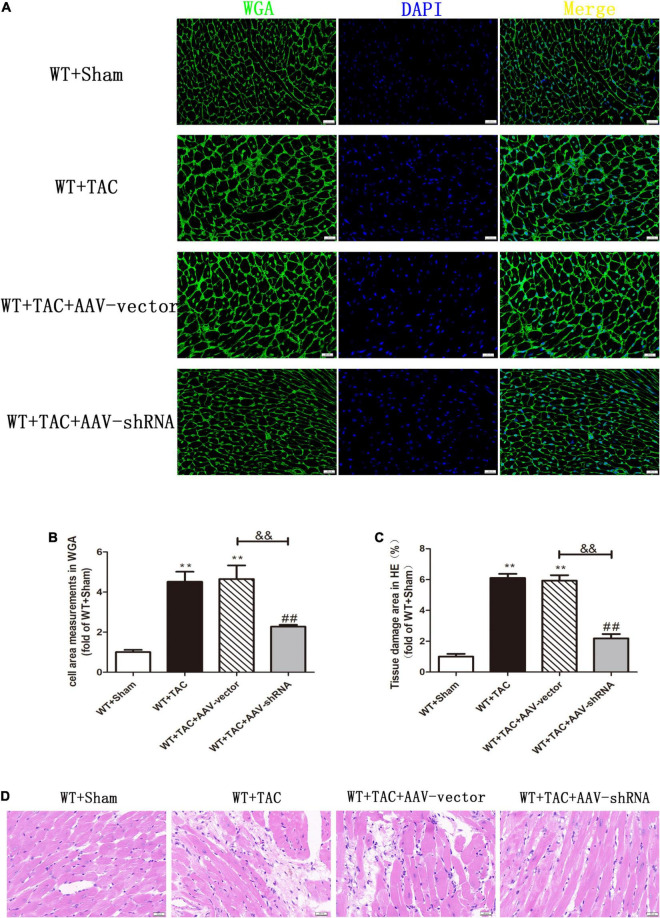
Interference with RIPK3 can inhibit the increase of cardiomyocyte cross-sectional area in HF mice and alleviate myocardial injury. Male 8-week-old WT mice were pretreated with AAV-vector and AAV-shRNA through the tail vein, and TAC was performed using a 28-g needle. The control group received sham surgery. All groups of mice were given continuous feeding for 6 weeks. Hearts were then obtained for correlative testing. **(A)** The cross-sectional area of mouse cardiomyocytes was observed by WGA staining, bar = 50 μm. **(B)** Cell area measurements in WGA staining. **(C)** Tissue damage area measurements in H&E staining. **(D)** H&E staining was used to detect the degree of myocardial injury, bar = 50 μm. Compared with WT + sham group, **P < 0.01; compared with WT + TAC group, ^##^*P* < 0.01; compared with WT + AAV-vector group, ^&&^*P* < 0.01. *n* = 6.

**FIGURE 11:**
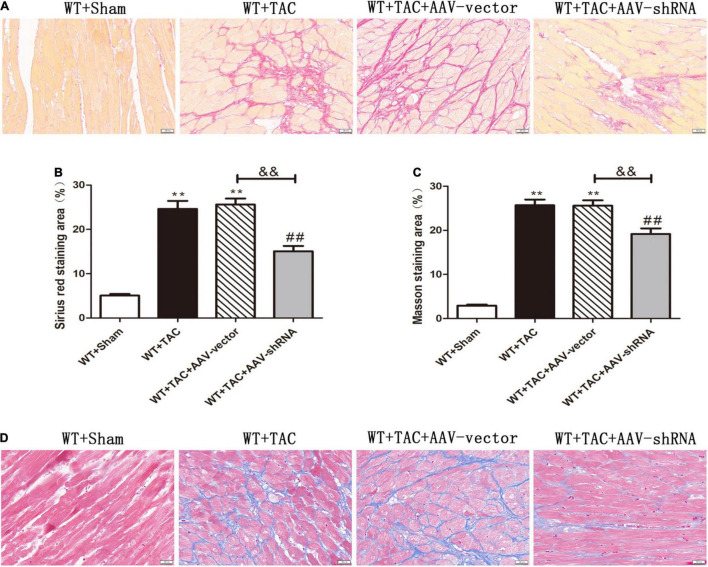
Interference with RIPK3 can improve the degree of collagen deposition and myocardial fibrosis in HF mice. Male 8-week-old WT mice were pretreated with AAV-vector and AAV-shRNA through the tail vein, and TAC was performed using a 28-g needle. The control group received sham surgery. All groups of mice were given continuous feeding for 6 weeks. Hearts were then obtained for correlative testing. **(A)** Sirius red staining, bar = 50 μm. **(B)** Sirius red staining area measurements. **(C)** Masson staining area measurements. **(D)** Masson staining, bar = 50 μm. Compared with WT + sham group, **P < 0.01; compared with WT + TAC group, ^##^*P* < 0.01; compared with WT + AAV-vector group, ^&&^*P* < 0.01. *n* = 6.

**FIGURE 12:**
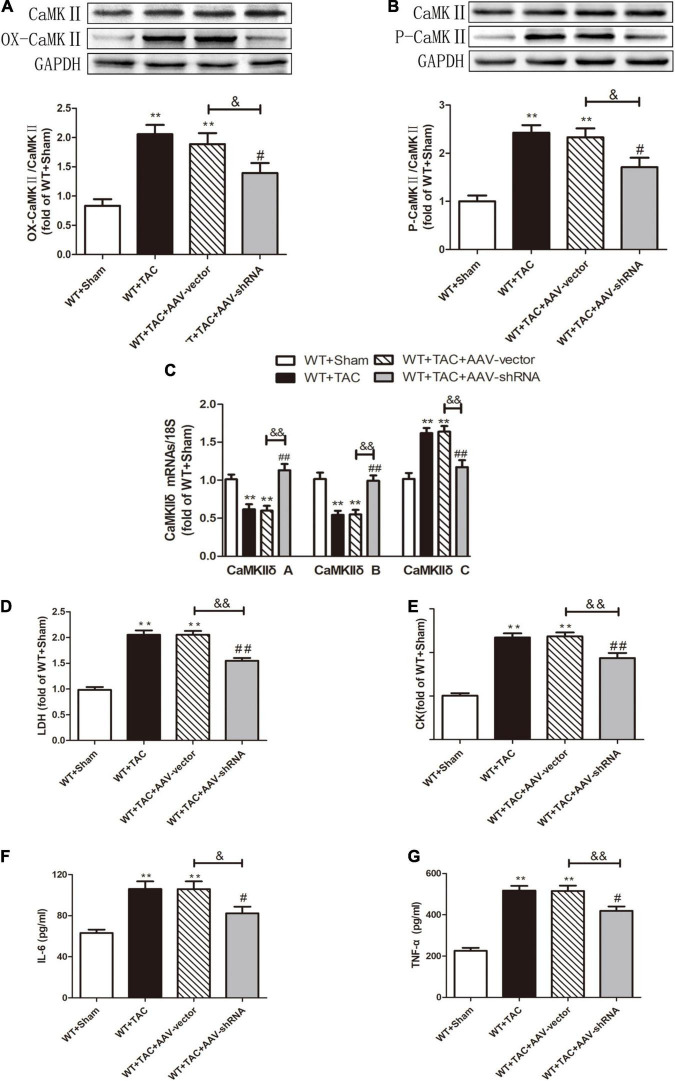
Interference with RIPK3 can alleviate the elevated levels of CaMKII oxidation and phosphorylation, improve CaMKIIδ alternative splicing disorder, the degree of myocardial injury, and inflammatory response in HF mice. Male 8-week-old WT mice were pretreated with AAV-vector and AAV-shRNA through the tail vein, and TAC was performed using a 28-g needle. The control group received sham surgery. All groups of mice were given continuous feeding for 6 weeks. Hearts were then obtained for correlative testing. **(A)** The expression of OX-CaMKII at the protein level in myocardial tissue was detected by Western blotting analysis, and GAPDH was used as a loading control. **(B)** The expression of P-CaMKII at the protein level in myocardial tissue was detected by Western blotting analysis, and GAPDH was used as a loading control. **(C)** Detection of myocardial CaMKIIδ A, CaMKIIδ B, and CaMKIIδ C at the mRNA level by qRT-PCR, and 18S was used as the housekeeping gene. **(D)** The levels of serum LDH, **(E)** CK, **(F)** IL-6, and **(G)** TNF-α. Compared with WT + sham group, **P < 0.01; compared with WT + TAC group, ^##^*P* < 0.01 and ^#^*P* < 0.05; compared with WT + AAV-vector group, ^&⁣&^*P* < 0.01 and ^&^*P* < 0.05. *n* = 6.

To further evaluate the relative role of RIPK3 in myocardial hypertrophy, we injected recombinant AAV-carrying RIPK3 shRNA directly into the tail vein of mice to interfere with the expression of RIPK3. TAC operation was performed on the pretreated mice, and the control group received sham surgery. Echocardiography showed no significant changes in IVS and LVPW in HF mice after interfering with the expression of RIPK3, while the EF and FS values of this group were increased significantly (Figures 9A–C), suggesting that the systolic cardiac function was improved. The expressions of hypertrophy and HF-related genes (ANP and BNP) in TAC-treated mice were increased significantly (Figures 9D,E). In addition, the cross-sectional area of cardiomyocytes in the model group was increased significantly, while the model group interfered with AAV-RIPK3 shRNA was improved significantly (Figures 10A,B). Meanwhile, H&E staining showed that after interfering with AAV-RIPK3 shRNA, the distortion and disorder of cardiomyocytes were reversed (Figures 10C,D). Sirius red staining (Figures 11A,B) and Masson staining (Figures 11C,D) showed that after interfering with AAV-RIPK3 shRNA, the deposition of central muscle collagen in HF mice was reduced, and the situation of myocardial fibrosis was improved. The levels of CK, LDH, IL-6, and TNF-α in mice were detected by corresponding kits (Figures 12D–G), and we found that myocardial tissue injury and inflammatory response were also reversed, indicating that interference with AAV-RIPK3 shRNA could indeed reduce myocardial injury in HF mice.

### AAV-RIPK3-shRNA Interferes With RIPK3 Expression, Alleviates Myocardial Necroptosis, Regulates CaMKIIδ Alternative Splicing Disorder in Heart Failure Mice, and Improves Oxidative Stress and Myocardial Mitochondrial Ultrastructure

**FIGURE 13:**
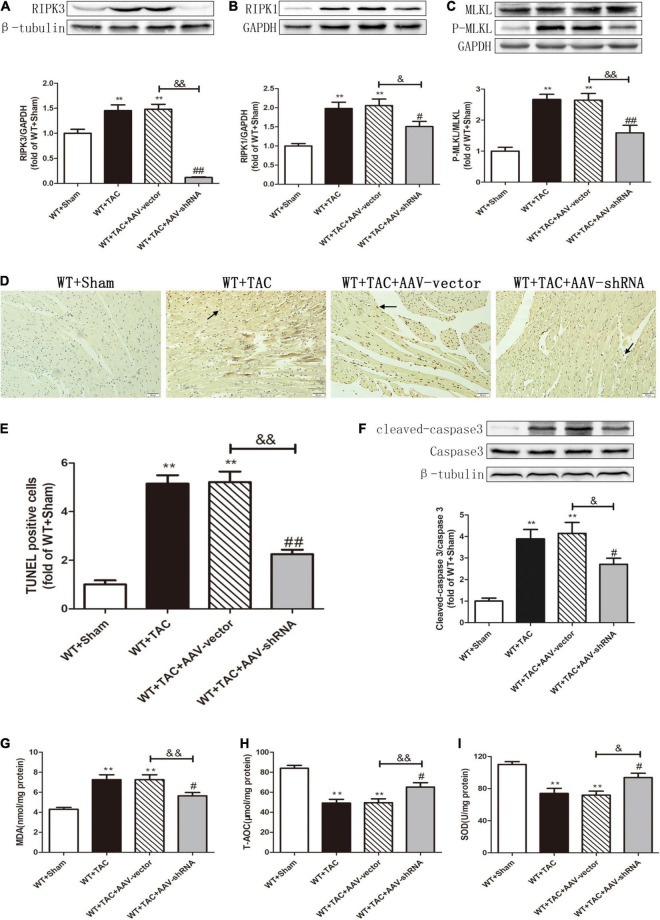
Interference with RIPK3 can alleviate the up-regulation of RIPK3 and RIPK1, as well as the MLKL phosphorylation, reduce cardiomyocyte necroptosis, and improve antioxidant capacity in HF mice. Male 8-week-old WT mice were pretreated with AAV-vector and AAV-shRNA through the tail vein, and TAC was performed using a 28-g needle. The control group received sham surgery. All groups of mice were given continuous feeding for 6 weeks. Hearts were then obtained for correlative testing. **(A)** The expression of RIPK3 at the protein level in myocardial tissue was detected by Western blotting analysis, and β-tubulin was used as a loading control. **(B)** The expression of RIPK1 at the protein level in myocardial tissue was detected by Western blotting analysis, and GAPDH was used as a loading control. **(C)** The expressions of MLKL and P-MLKL at the protein level in myocardial tissue were detected by Western blotting analysis, and GAPDH was used as a loading control. **(D)** The level of apoptosis in myocardial tissue was measured by TUNEL staining, bar = 50 μm. **(E)** The statistical chart of TUNEL-positive cells. **(F)** The expressions of cleaved caspase 3 and caspase 3 at the protein level in myocardial tissue were detected by Western blotting analysis, and β-tubulin was used as a loading control. **(G)** The levels of serum MDA, **(H)** T-AOC, **(I)** and SOD. Compared with WT + sham group, **P < 0.01; compared with WT + TAC group, ^##^*P* < 0.01 and ^#^*P* < 0.05; compared with WT + AAV-vector group, ^&⁣&^*P* < 0.01 and ^&^*P* < 0.05. *n* = 6.

**FIGURE 14:**
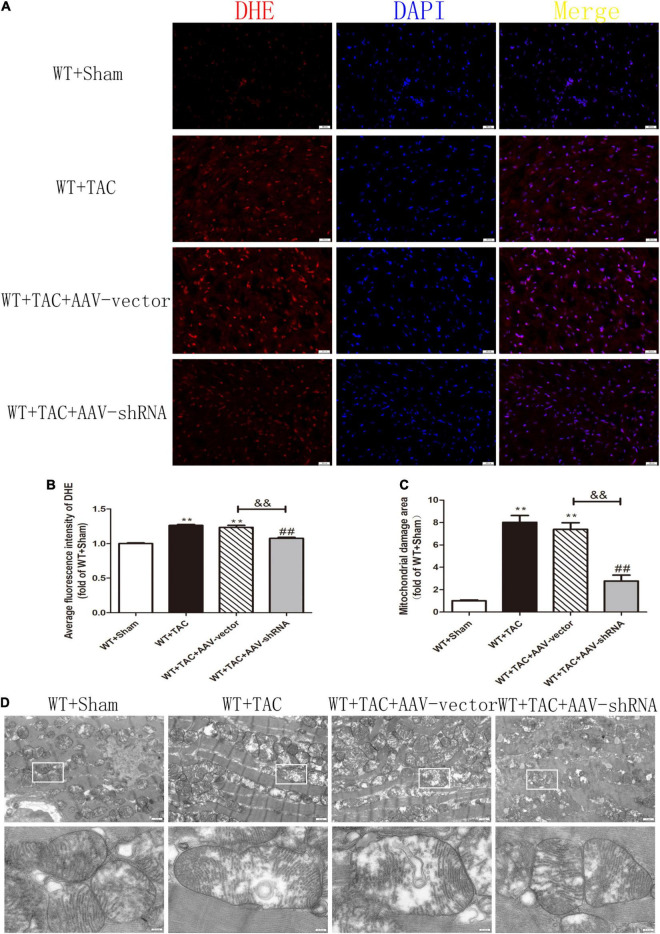
Interference with RIPK3 can reduce ROS accumulation in myocardial tissue of HF mice and improve antioxidant capacity and mitochondrial structural abnormalities. Male 8-week-old WT mice were pretreated with AAV-vector and AAV-shRNA through the tail vein, and TAC was performed using a 28-g needle. The control group received sham surgery. All groups of mice were given continuous feeding for 6 weeks. Hearts were then obtained for correlative testing. **(A)** DHE staining, bar = 50 μm. **(B)** The average fluorescence intensity measurements of DHE staining. **(C)** Mitochondrial damage area. **(D)** The ultrastructure of LV mitochondria was observed by transmission electron microscope, bar = 2 μm. Lower picture bar = 0.5 μm. Compared with WT + Sham group, **P < 0.01; compared with WT + TAC group, ^##^*P* < 0.01; compared with WT + AAV-vector group, ^&⁣&^*P* < 0.01. *n* = 6.

After injection of the recombinant AAV through the caudal vein, the oxidation and phosphorylation levels of its downstream necroptosis-related effector CaMKII were inhibited (Figures 12A,B). In addition, the alternative splicing disorder of CaMKIIδ was corrected (Figure 12C). Moreover, due to interference with the expression of RIPK3, the up-regulation of RIPK3 in HF mice was inhibited (Figure 13A). In HF mice, after injection of AAV, the expression of RIPK1, a downstream related protein of RIPK3, and the phosphorylation of MLKL were inhibited (Figures 13B,C). TUNEL staining and the expression of cleaved caspase 3 showed improved apoptosis (Figures 13D–F). In addition, the ability of myocardial tissue to scavenge oxygen free radicals was enhanced, and ROS accumulation was reduced (Figures 13G–I). Besides, DHE staining (Figures 14A,B) and transmission electron microscopy (Figures 14C,D) showed that after RIPK3 interference, the oxidative stress level and mitochondrial ultrastructural abnormalities in HF mice were improved.

## Discussion

Many studies have shown that the heart structure and function of HF are changed. The typical manifestations of HF are myocardial fibrosis, arrhythmia, and hypertrophy. Our results showed that the E/A was significantly decreased, and the expressions of ANP and BNP were increased in HF mice. The incidence rate and mortality rate of HF are high worldwide, while the prevention and treatment methods remain very limited (29). Necroptosis is regulated necrosis that can lead to cardiovascular diseases, such as vascular atherosclerosis, ischemia/reperfusion injury (IRI), myocardial infarction, and cardiac remodeling (28). Necroptosis is a particular form of apoptosis. In our present study, TUNEL staining and the expression of cleaved caspase 3 revealed severe apoptosis in HF mice. Meanwhile, the expressions of RIPK3, RIPK1, and P-MLKL, which mediate necroptosis, were also significantly increased, indicating no apoptosis. However, instead, necroptosis occurred here. In addition, RIPK3 was also involved in myocardial injury in the process of HF, while depletion of RIPK3 could reduce cardiac dysfunction, myocardial injury, and necroptosis in HF mice. This finding was further confirmed in AAV-RIPK3-shRNA-infected mice.

RIPK1 and RIPK3 belong to the same family. When caspase 8 is inhibited or defective, RIPK1 will combine with RIPK3 through the C-terminal rip homologous interaction motif (RHIM) domain to form a RIPK1/RIPK3 complex, called “necrotic body.” The complex initiates downstream signal transduction and triggers necroptosis (5, 30). Excessive necroptosis can lead to embryo death (31) and a variety of heart diseases, including but not limited to myocardial infarction, IRI, and HF (32). MLKL is a pseudokinase produced by RIPK1 binding and phosphorylating RIPK3, and then phosphorylated RIPK3 undergoes further phosphorylation, which is called MLKL (33). Then, phosphorylated MLKL (P-MLKL) is transferred to the plasma membrane and oligomerized to form pores on the cell membrane, thereby increasing the permeability of the cell membrane (34) and leading to cell expansion and final rupture (35). Recent studies have shown that necrotic bodies formed by RIPK3 and P-MLKL oligomers are essential for necroptosis (36). Our results showed that the RIPK1 expression and MLKL phosphorylation were significantly up-regulated in the myocardium of HF mice, indicating the existence of necroptosis, and MLKL pathway might be the downstream or substrate of RIPK3-mediated necroptosis. This finding was significantly alleviated in RIPK3^–/–^ mice with HF. Moreover, this was also confirmed in AAV-RIPK3-shRNA infected mice. However, the specific pathway of RIPK3-induced myocardial necroptosis is still unclear.

Caspases are a group of cysteine proteases that can regulate apoptosis. Caspases cleave substrates at the carboxyl end of specific aspartic acid residues. These proteases usually exist in healthy cells as inactive enzymes, and they will be activated by autolytic division when stimulated. These enzymes then cleave the substrate at one or two specific sites, leading to the activation, inactivation, relocation, or remodeling of the substrate (37). The activation of caspase 3 is necessary for most morphological and biochemical events related to apoptosis (38, 39), and caspase 3 is responsible for most protein hydrolysis in apoptosis (40). The cleavage of caspase 3 is usually considered a universal marker of apoptosis. Our study found that the expression of cleaved caspase 3 was increased significantly in the heart tissue of HF mice, suggesting apoptosis. As mentioned above, the expressions of RIPK3 and downstream RIPK1 and P-MLKL, which mediate necroptosis, were increased, and necroptosis is a particular type of apoptosis. Therefore, we believed that the apoptosis here was necroptosis. In RIPK3^–/–^ mice with HF, the expression of cleaved caspase 3 was significantly lower compared with the control group, and the same result was observed in AAV-RIPK3-shRNA-infected mice, indicating that depletion or inhibition of RIPK3 could effectively reduce the occurrence of myocardial necroptosis.

CaMKII is a pleiotropic signal molecule that regulates gene expression, contraction, metabolism, ROS, Ca^2+^ homeostasis, and inflammatory response of cardiomyocytes (41). CaMKII is inactivated under basal conditions. Continuous activation of CaMKII can promote cardiomyocyte death under endoplasmic reticulum (ER) stress, hyperglycemia, and IRI (42–44). It has been found that RIPK3 leads to myocardial necroptosis through oxidation and phosphorylation of CaMKII after IRI or adriamycin stimulation (9). Our study found that the phosphorylation and oxidation of CaMKII were significantly enhanced in cardiomyocytes of HF mice, while such up-regulation was significantly reversed in RIPK3^–/–^ and AAV-RIPK3-shRNA-infected mice. In conclusion, CaMKII signaling pathway might be the downstream or substrate of RIPK3-mediated myocardial injury and necroptosis in HF.

Many studies have confirmed that mitochondrial dysfunction is closely related to necroptosis. Mitochondria are the primary source of energy and ROS production, and mitochondrial dysfunction leads to excessive ROS production and cell death (45). Up-regulation of RIPK3 induces ER stress, accompanied by increased intracellular Ca^2+^ level and xanthine oxidase (XO) expression. Activated XO increases cellular ROS that mediates MPTP opening and cardiomyocyte necroptosis (46). Our study found that depletion or inhibition of RIPK3 improved the ultrastructure of myocardial mitochondria in HF mice. These results emphasized that mitochondrial damage might be the underlying mechanism of RIPK3-induced necroptosis in HF.

In conclusion, our study supported the presence of severe necroptosis in HF. Depletion or inhibition of RIPK3 could reduce myocardial injury, improve cardiac function, inhibit CaMKII activation, regulate CaMKIIδ alternative splicing disorder, down-regulate the expression of P-MLKL, and attenuate necroptosis, inflammatory response, and oxidative stress in HF mice. Collectively, myocardial injury and necroptosis caused by activation of CaMKII and MLKL were increased in HF in a RIPK3-dependent manner. These findings provided valuable insights into the pathological process and mechanism of HF and offered new treatment strategies for HF.

## Data Availability Statement

The original contributions presented in the study are included in the article/Supplementary Material, further inquiries can be directed to the corresponding authors.

## Ethics Statement

The animal study was reviewed and approved by all procedures were in accordance with the guidelines for the care and use of laboratory animals issued by the National Institutes of Health and the recommendations of the committee on the care and use of teaching animals of Nantong University (approval No.: NTU – 20161225).

## Author Contributions

JC and JZ were involved in the conception and design of the work, analysis of data, manuscript drafting, and contributed equally to this work. JQ and XW were involved in the acquisition and analysis of data. WZ and XC were involved in the design of the research report and proofreading of the original manuscript. All authors read and approved the final manuscript submitted for publication.

## Conflict of Interest

The authors declare that the research was conducted in the absence of any commercial or financial relationships that could be construed as a potential conflict of interest.

## Publisher’s Note

All claims expressed in this article are solely those of the authors and do not necessarily represent those of their affiliated organizations, or those of the publisher, the editors and the reviewers. Any product that may be evaluated in this article, or claim that may be made by its manufacturer, is not guaranteed or endorsed by the publisher.
