# Supplementary figures and images for: Ca2+/Calmodulin-Dependent Protein Kinase II Regulation by RIPK3 Alleviates Necroptosis in Transverse Arch Constriction-Induced Heart Failure
Source: Front Cardiovasc Med. 2022 Apr 28;9:847362. doi: 10.3389/fcvm.2022.847362 (PMC9097920; doi:10.3389/fcvm.2022.847362)

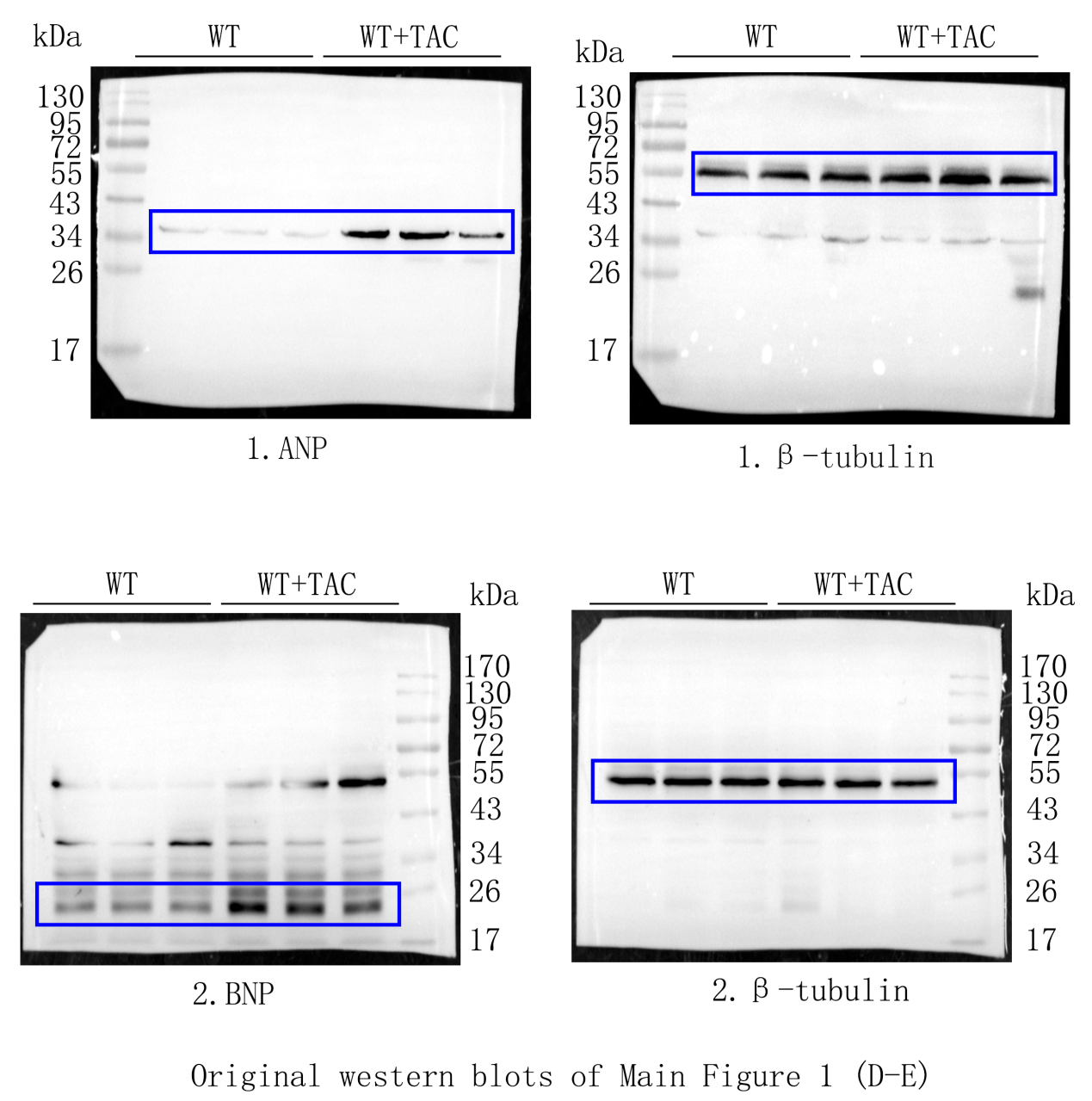


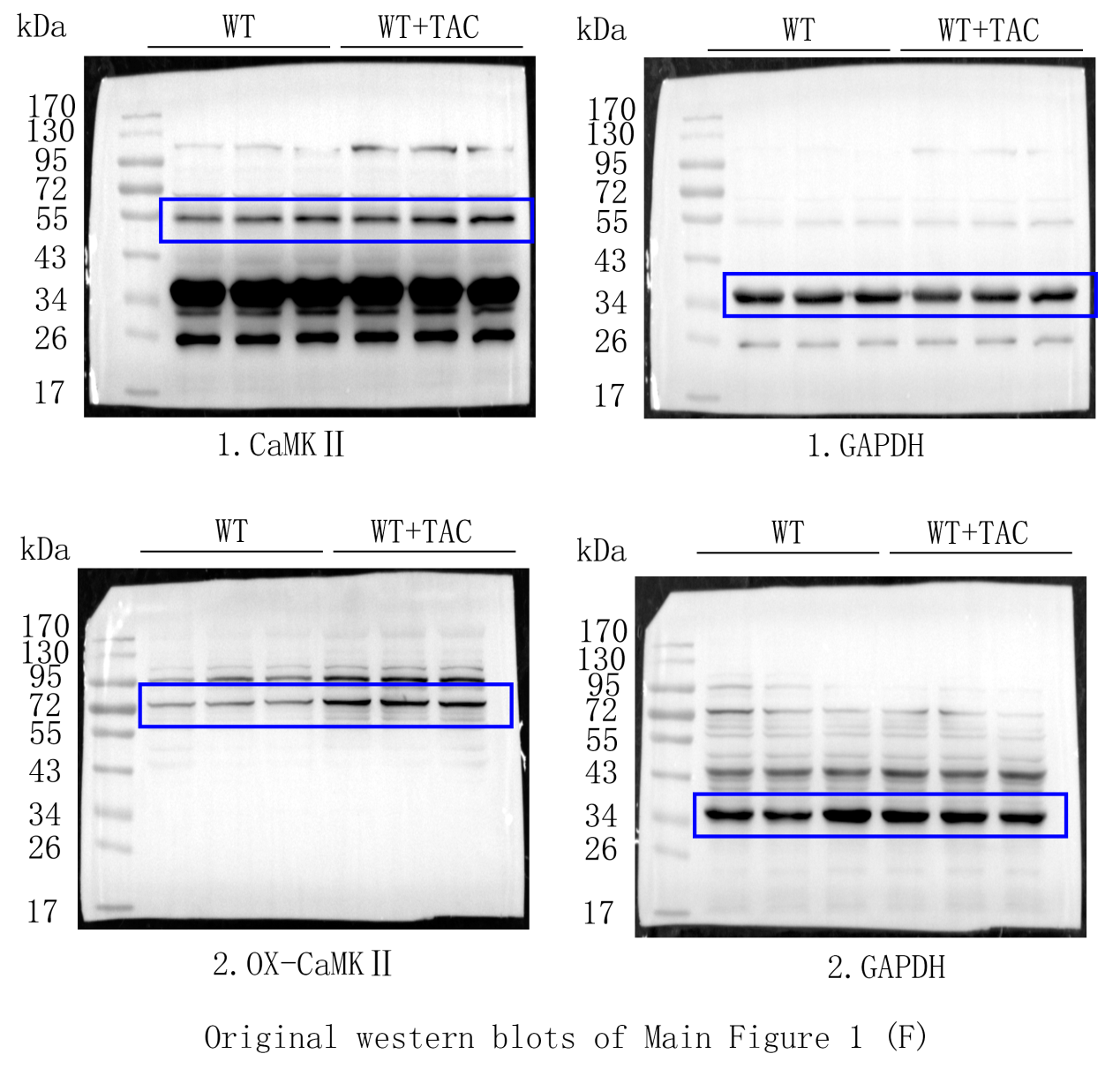


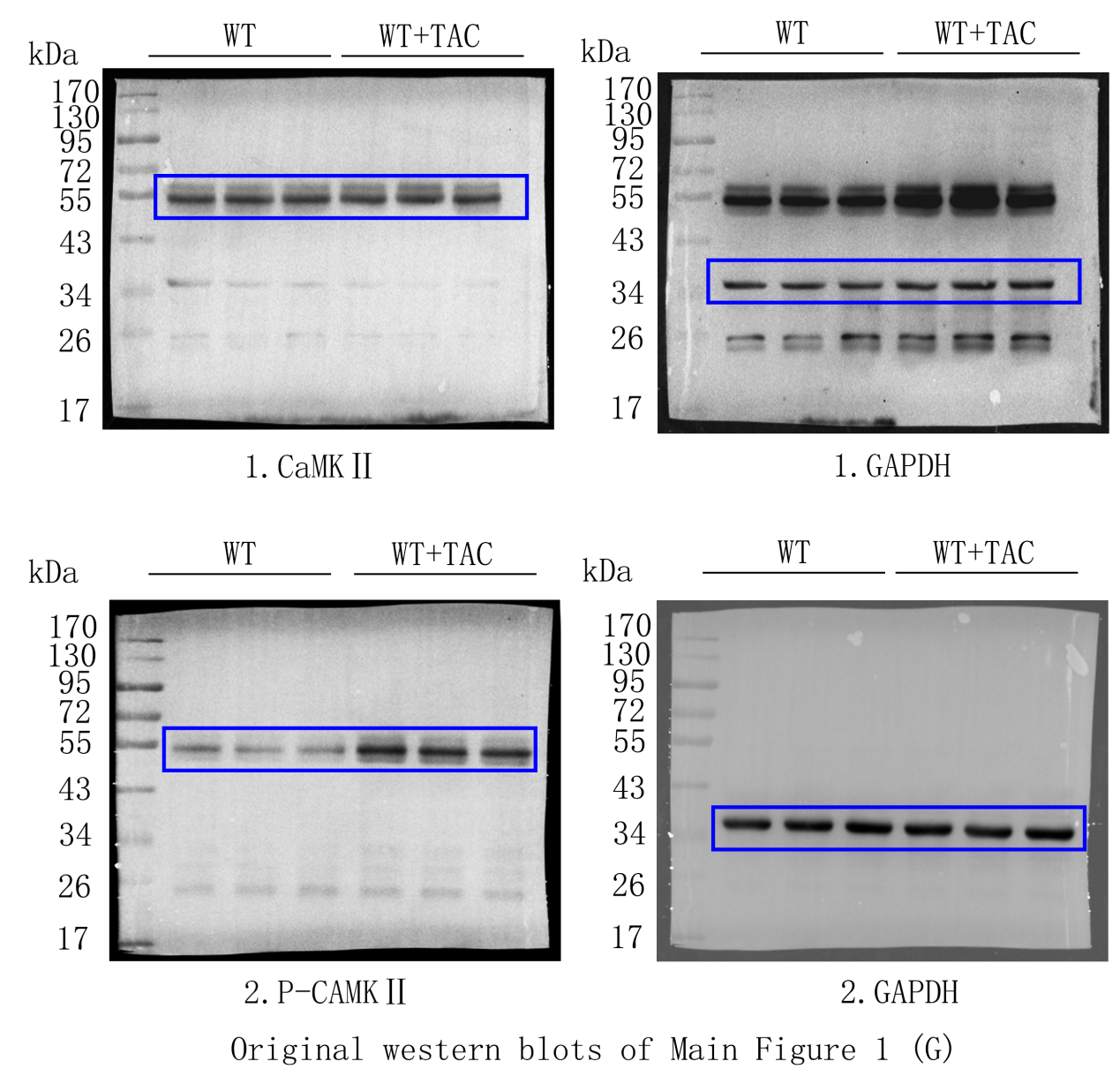

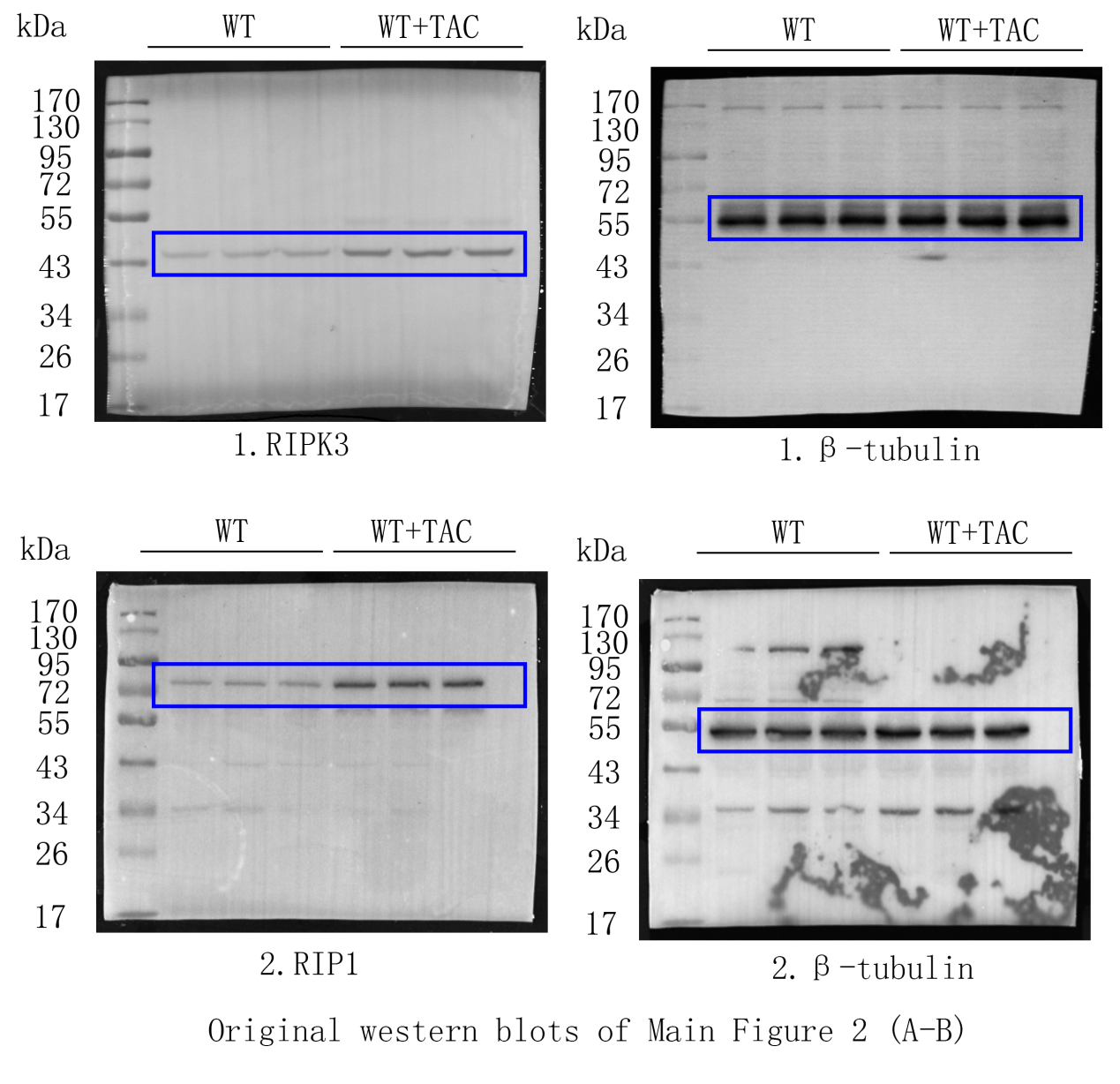

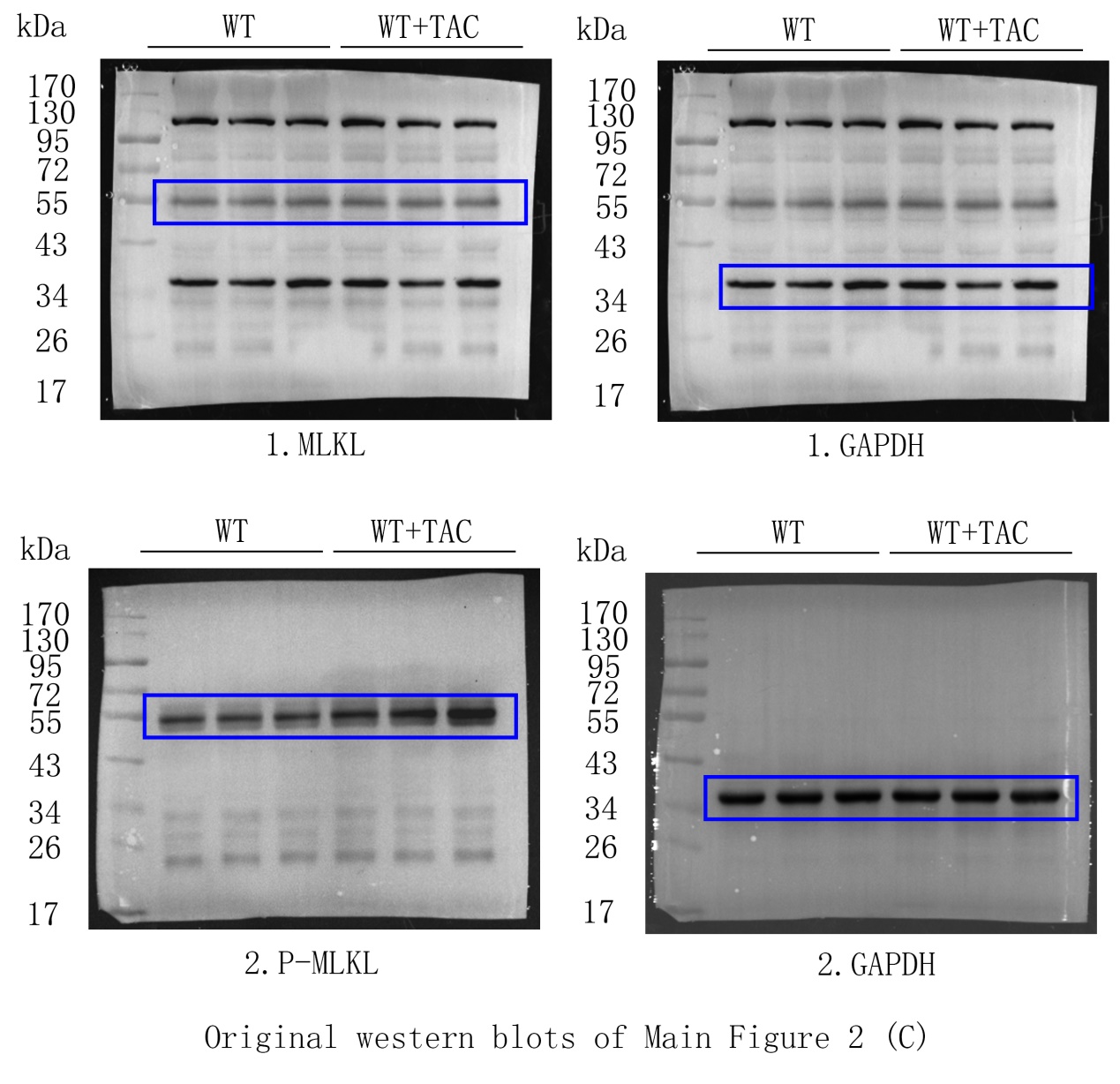

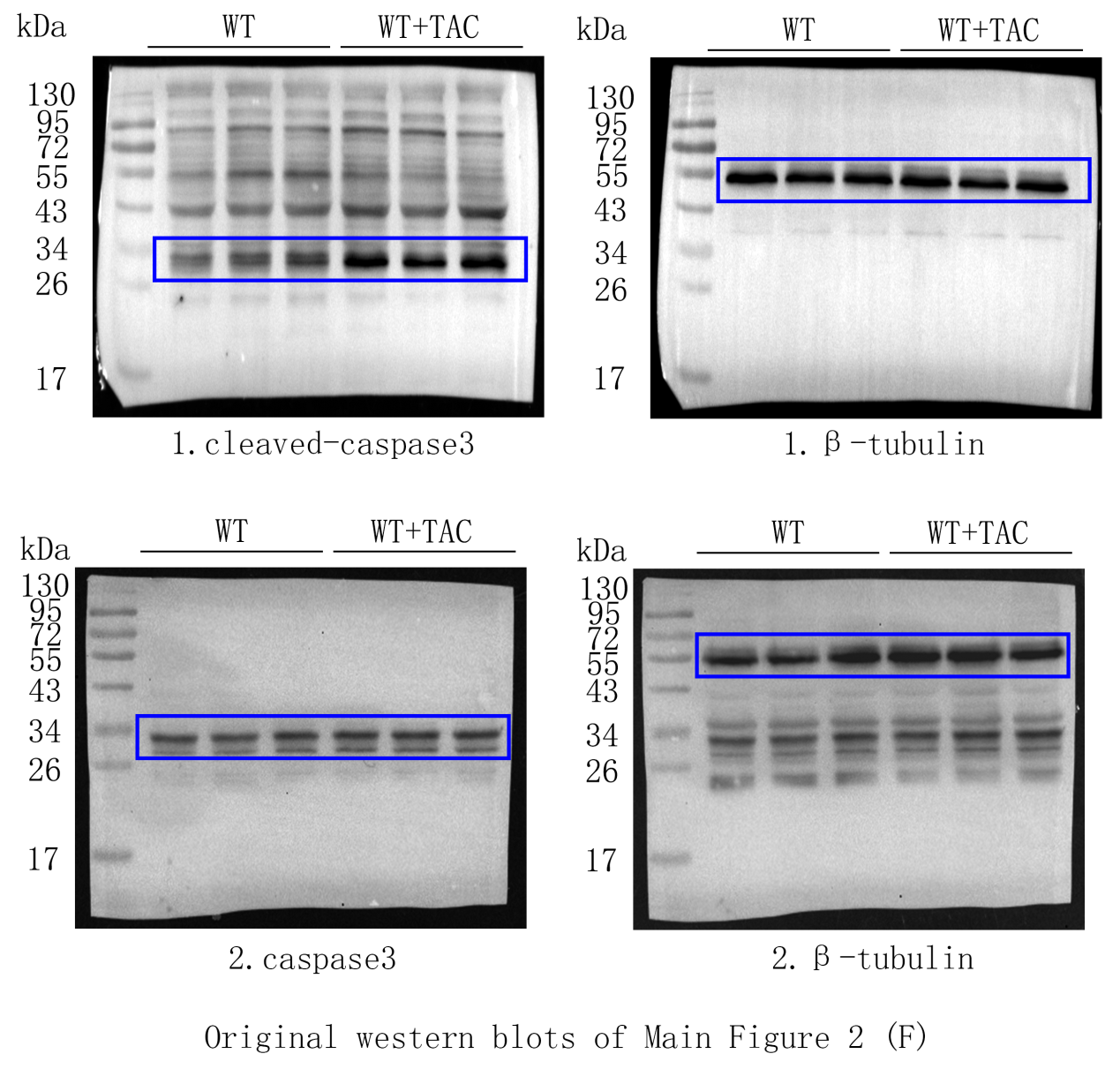

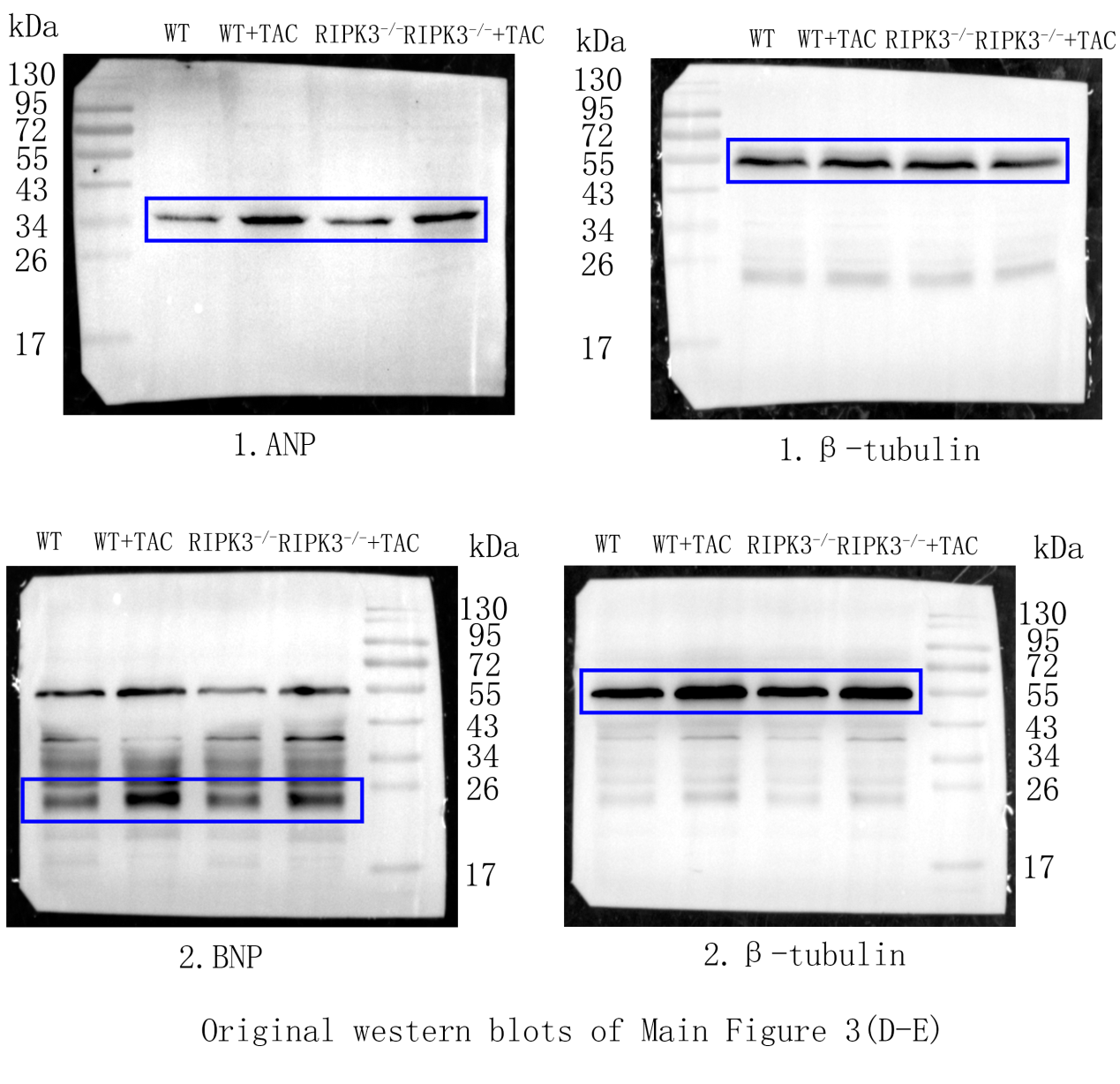

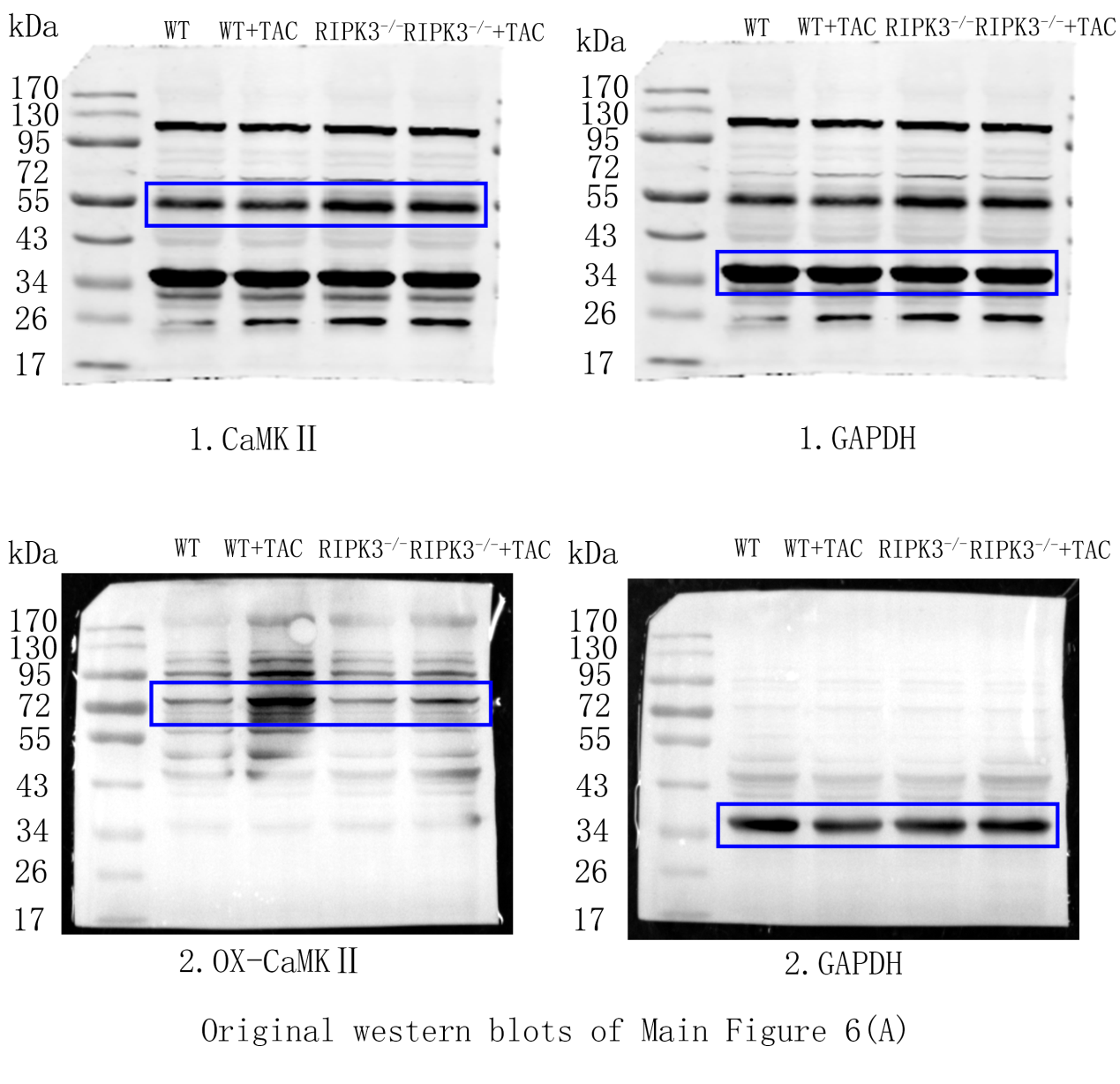

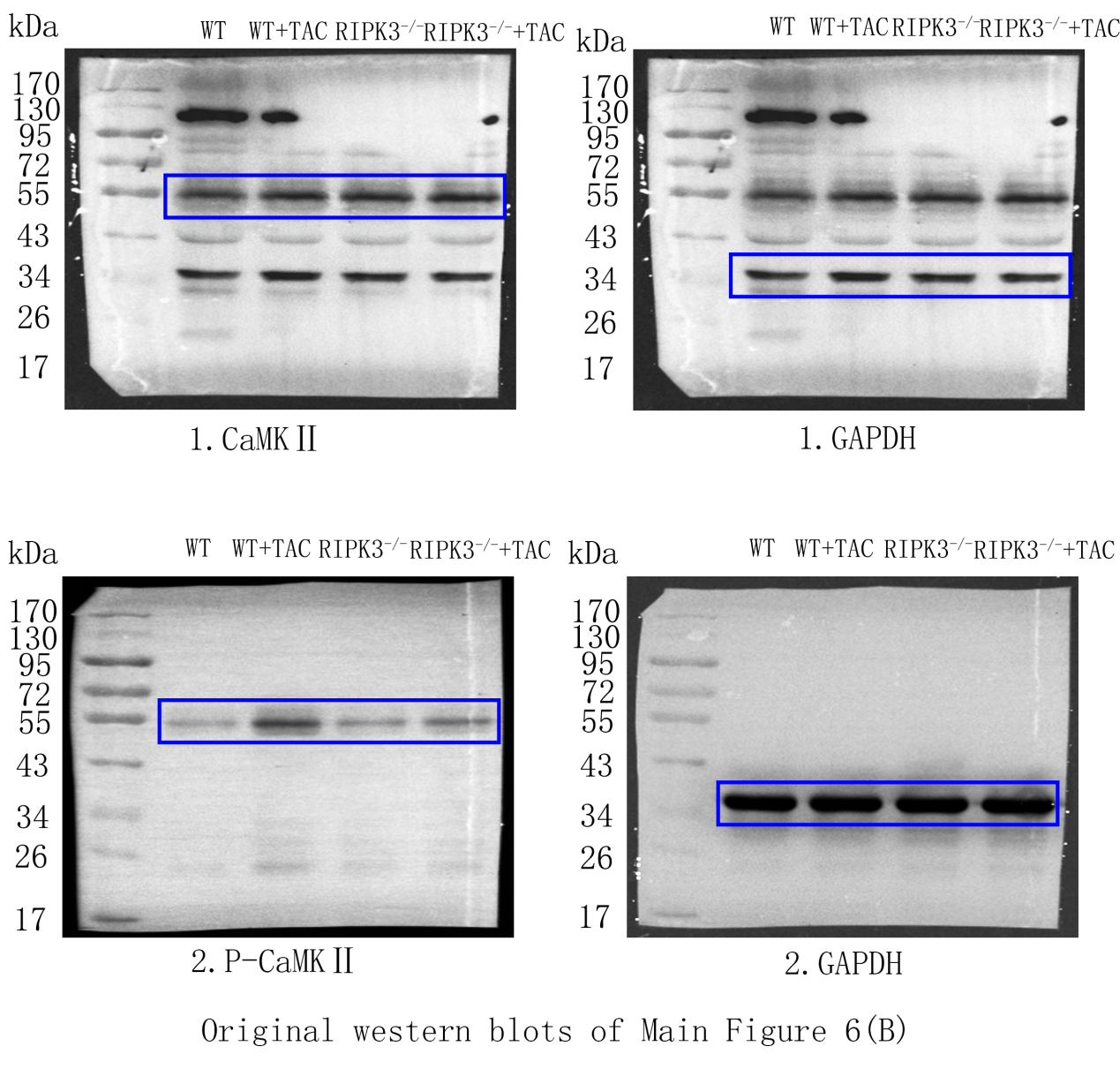

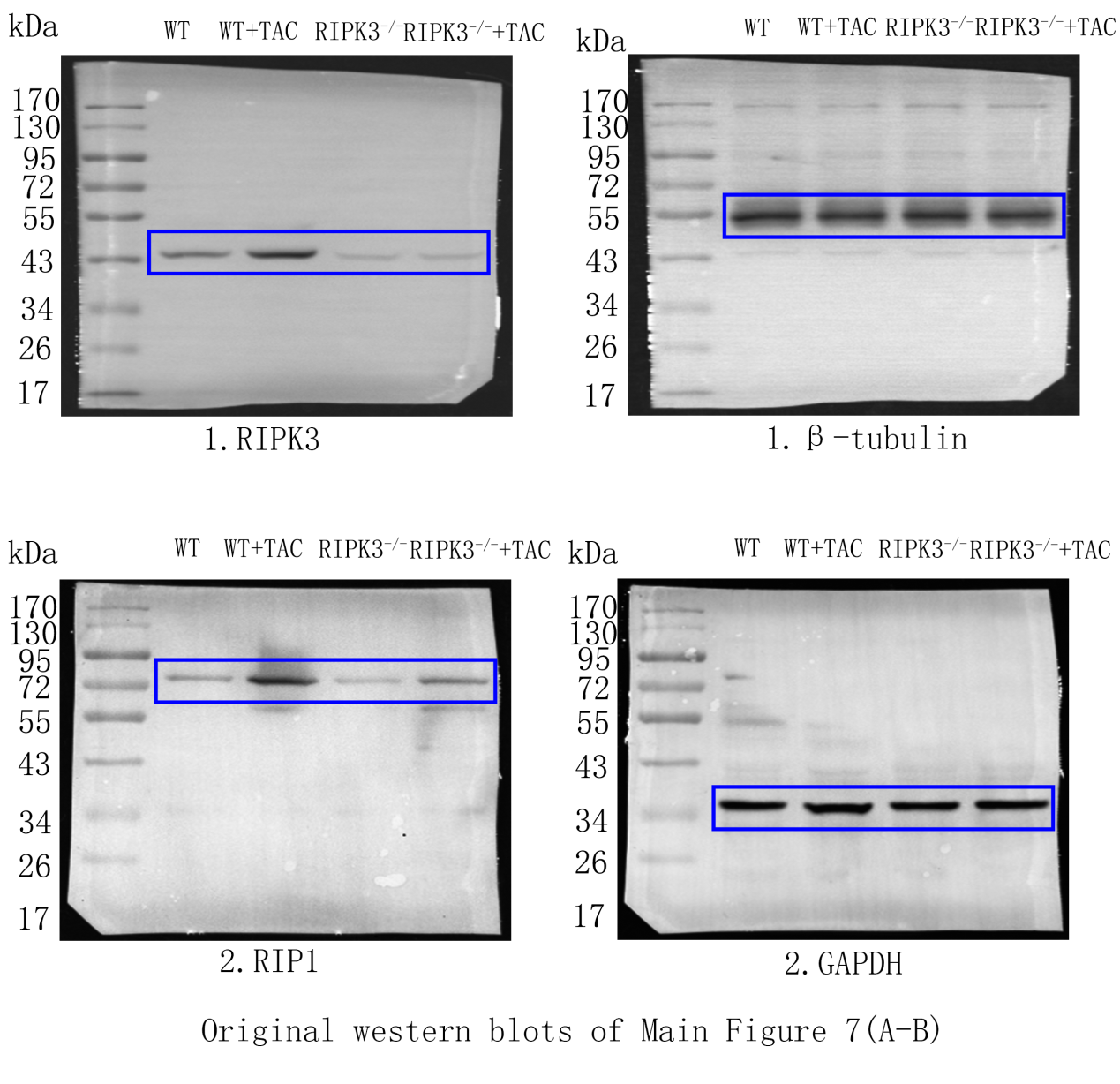

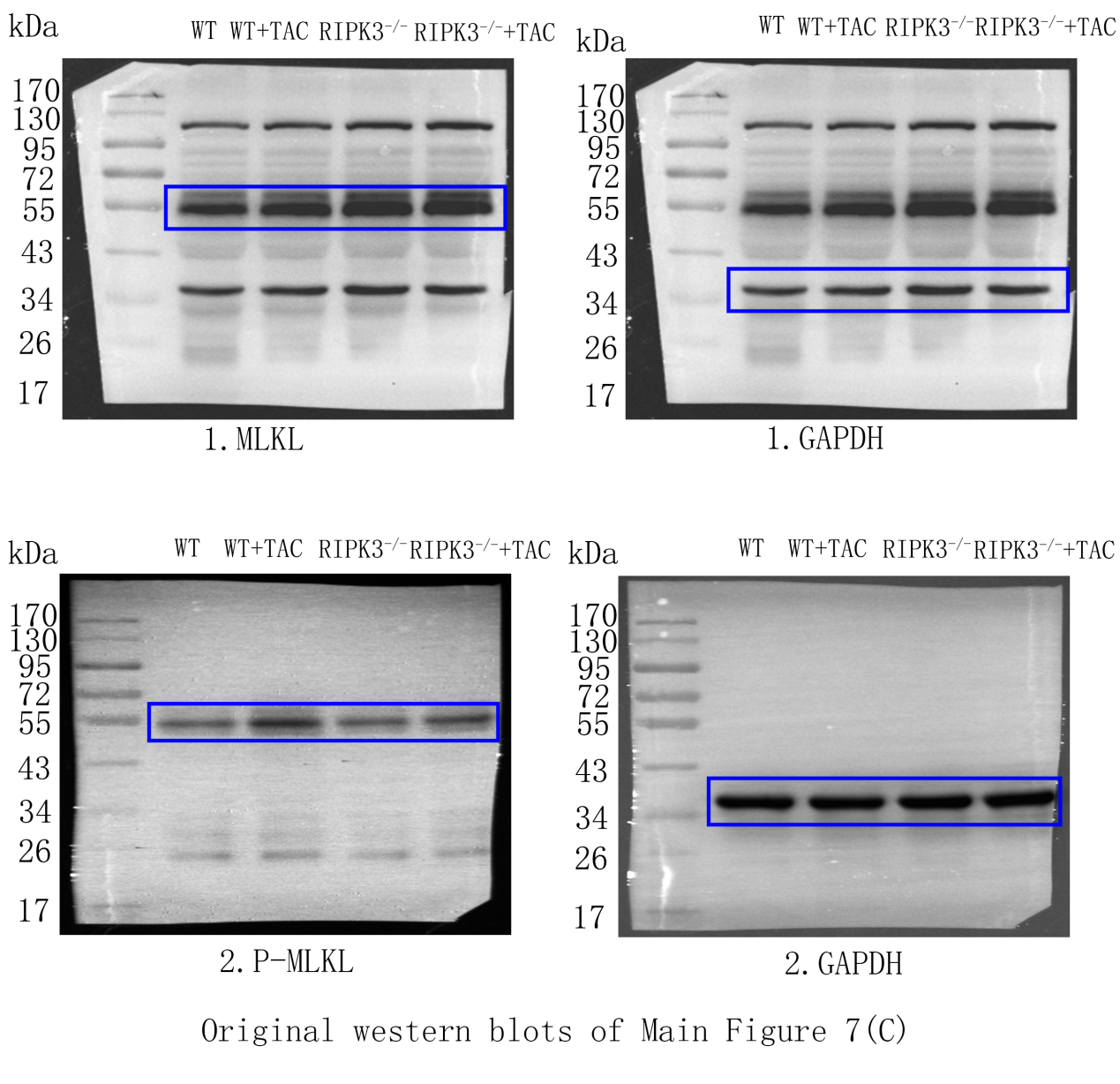

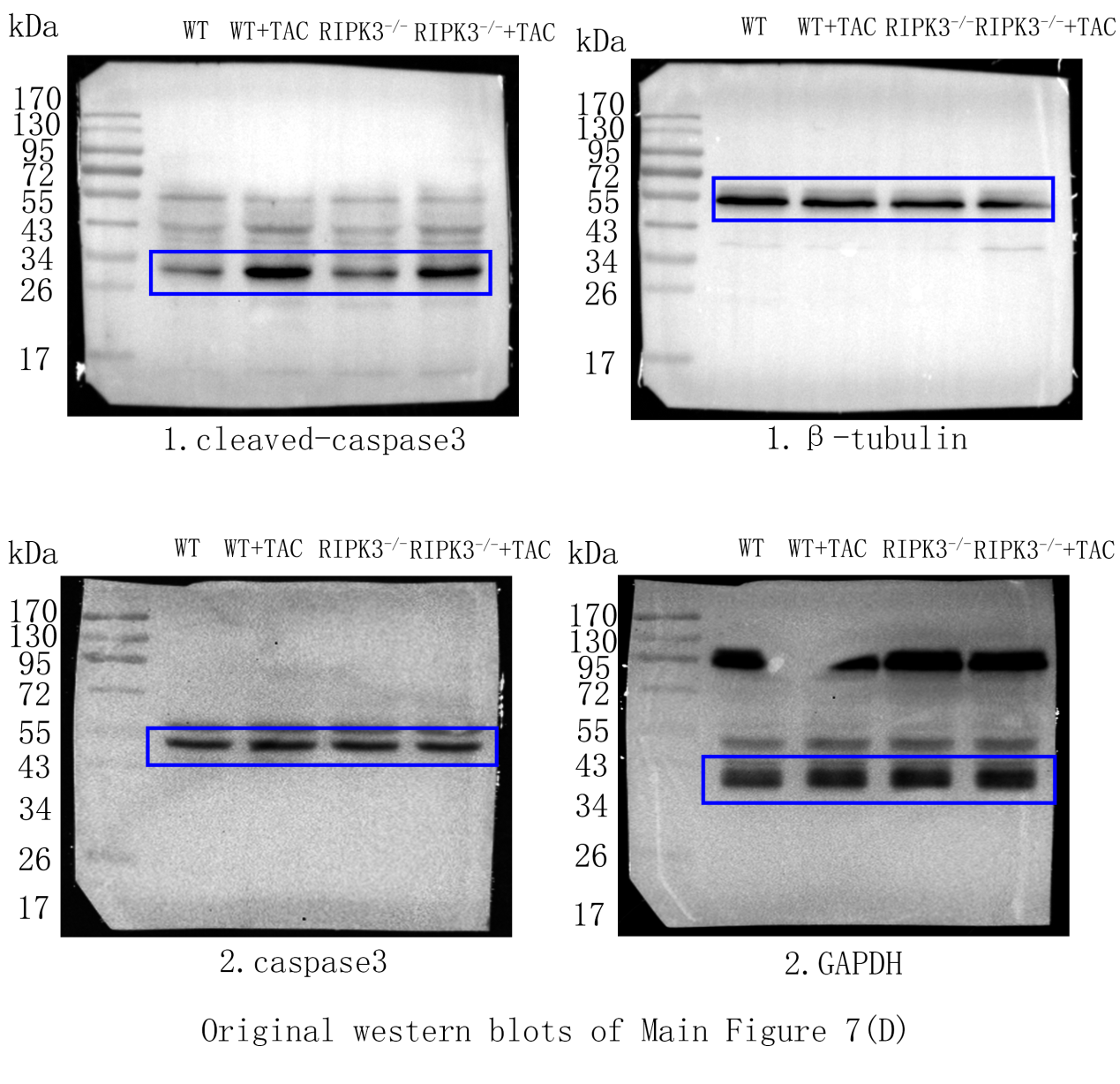

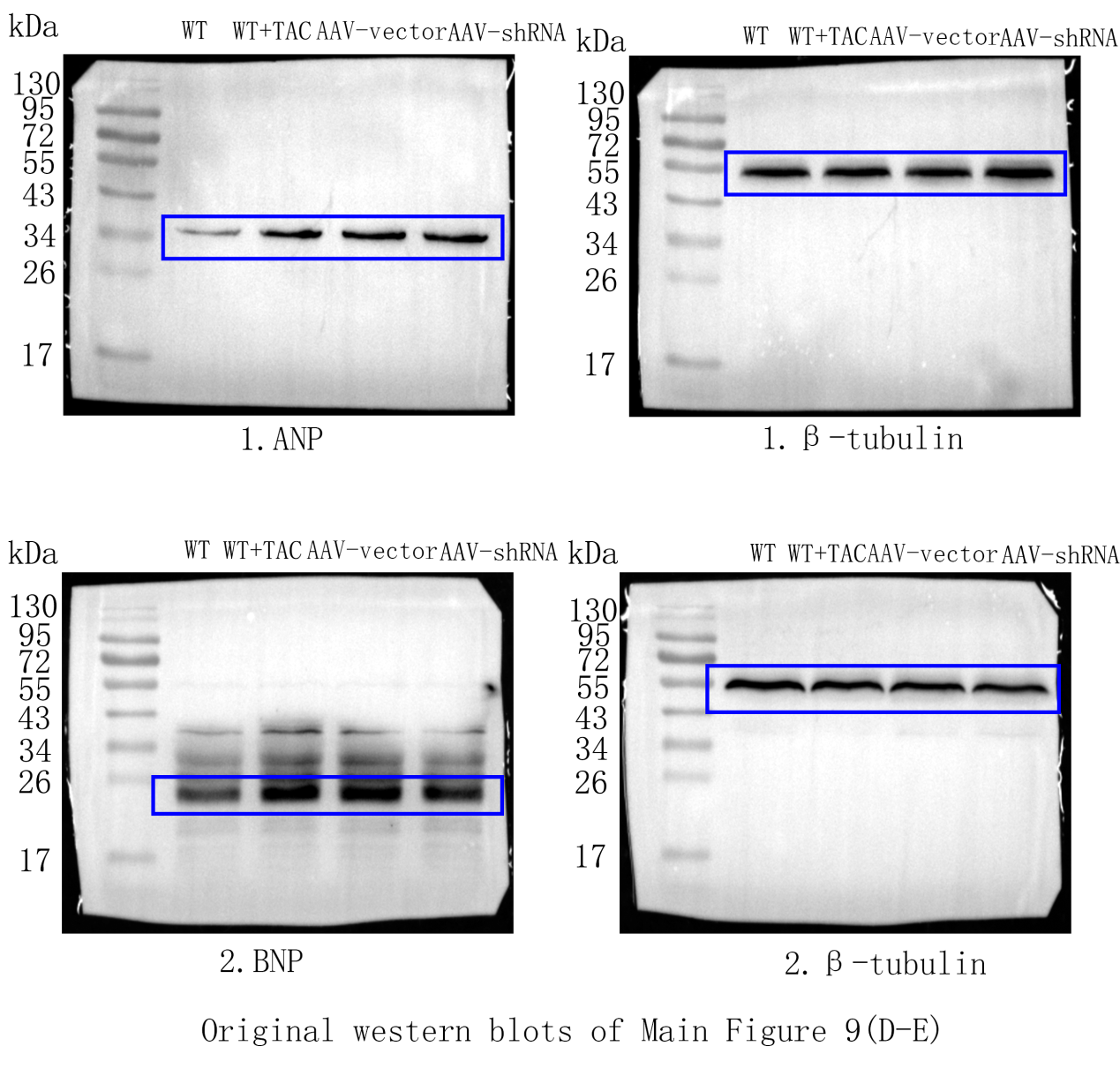

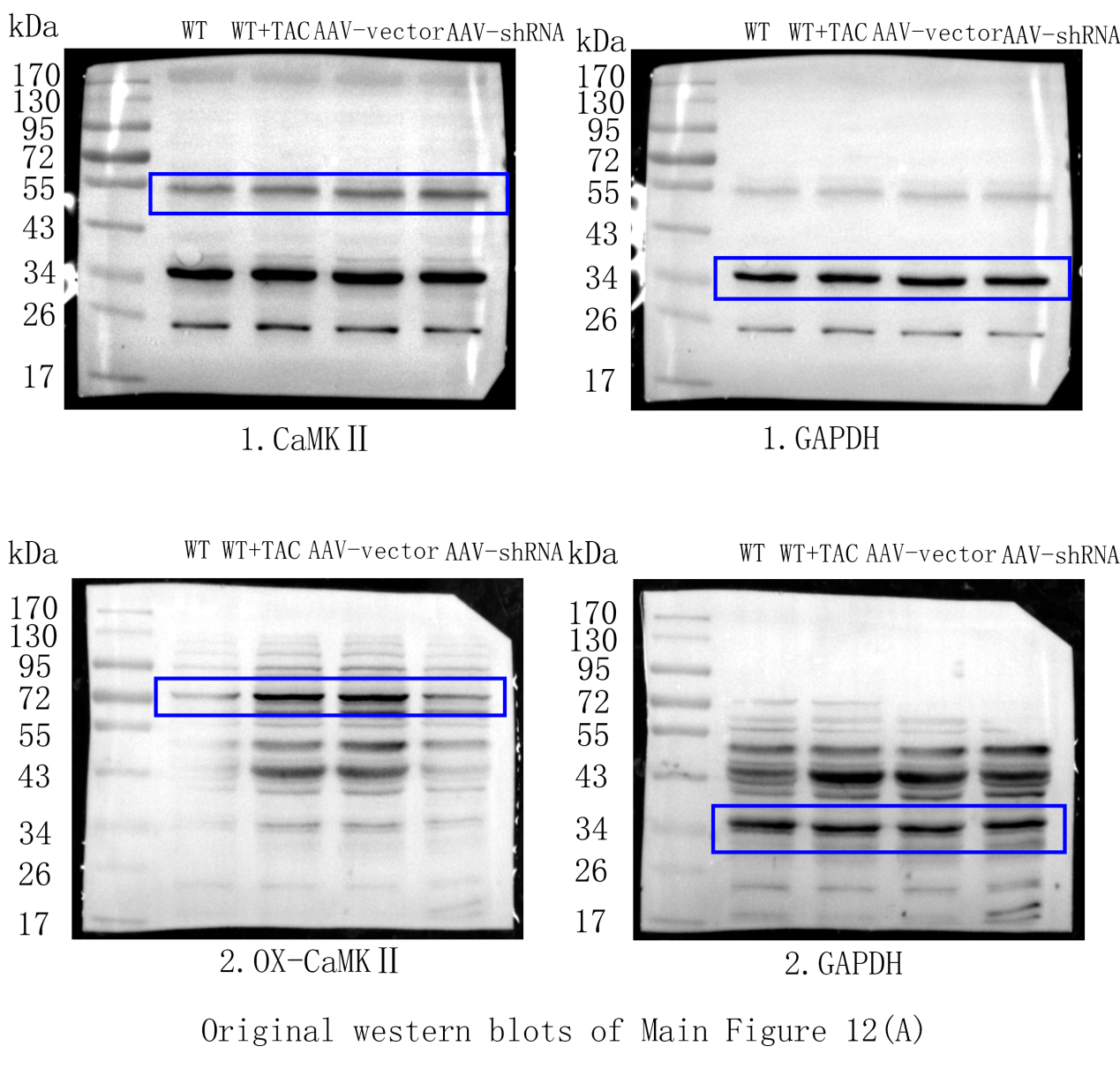

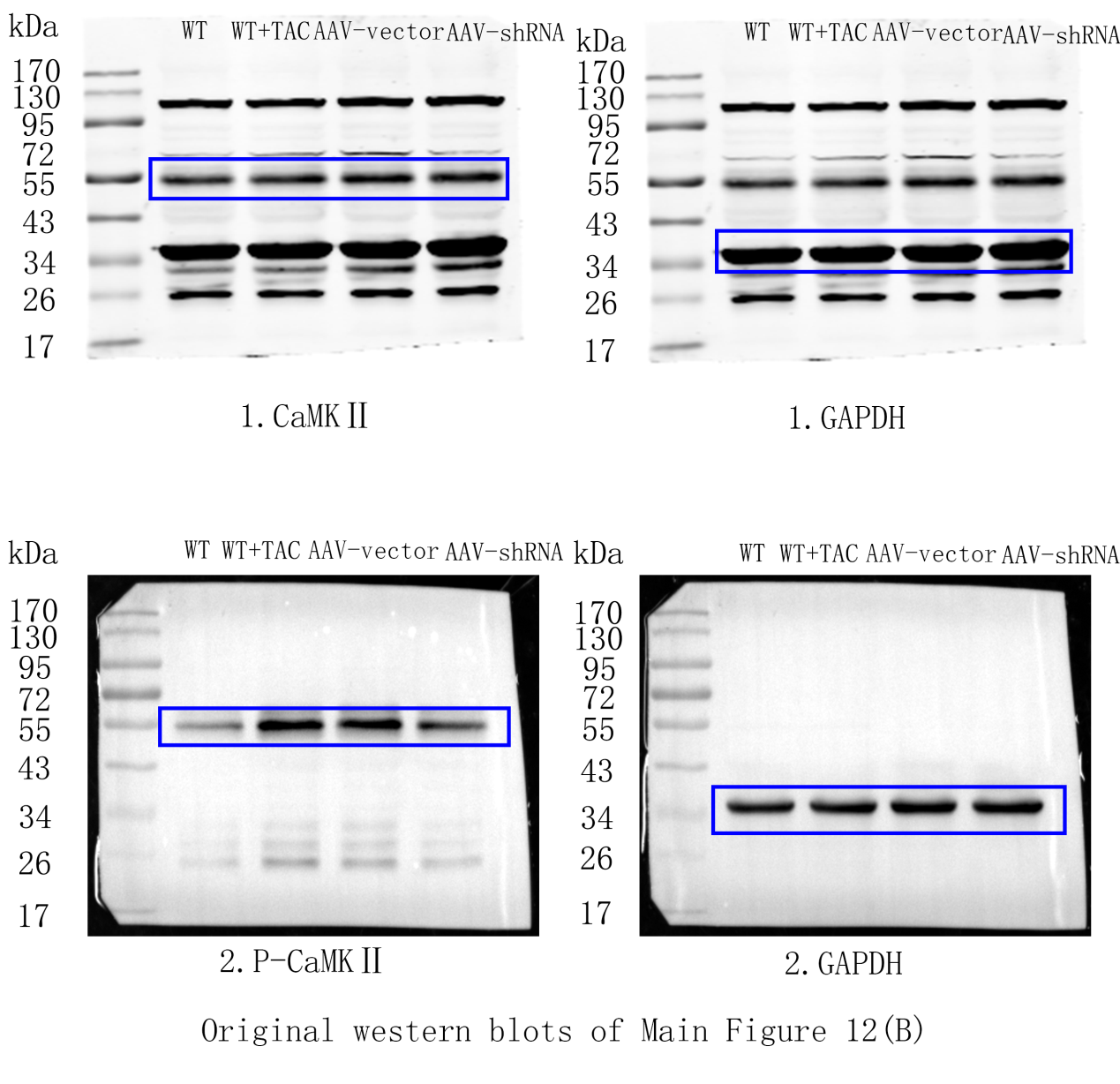

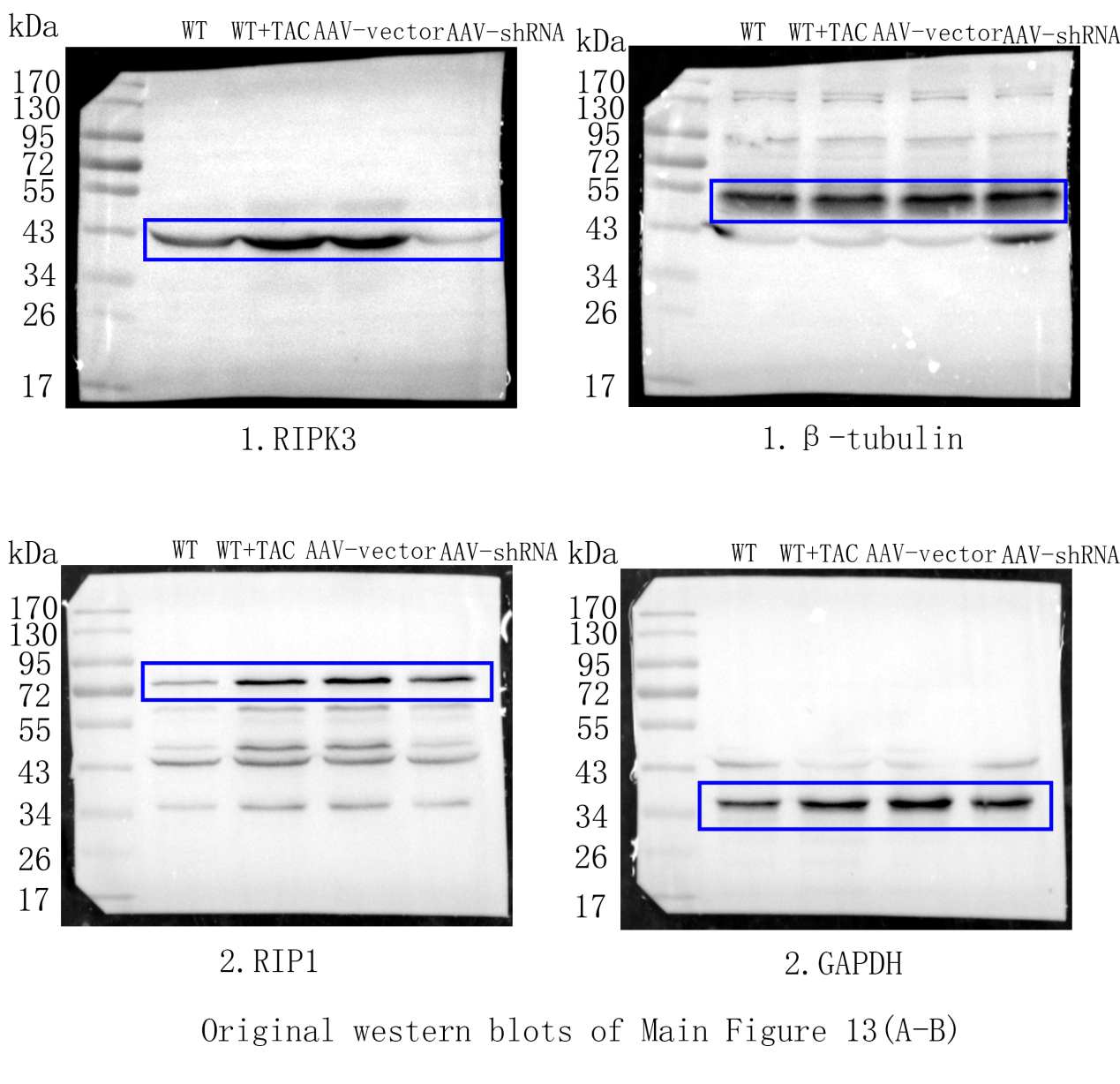

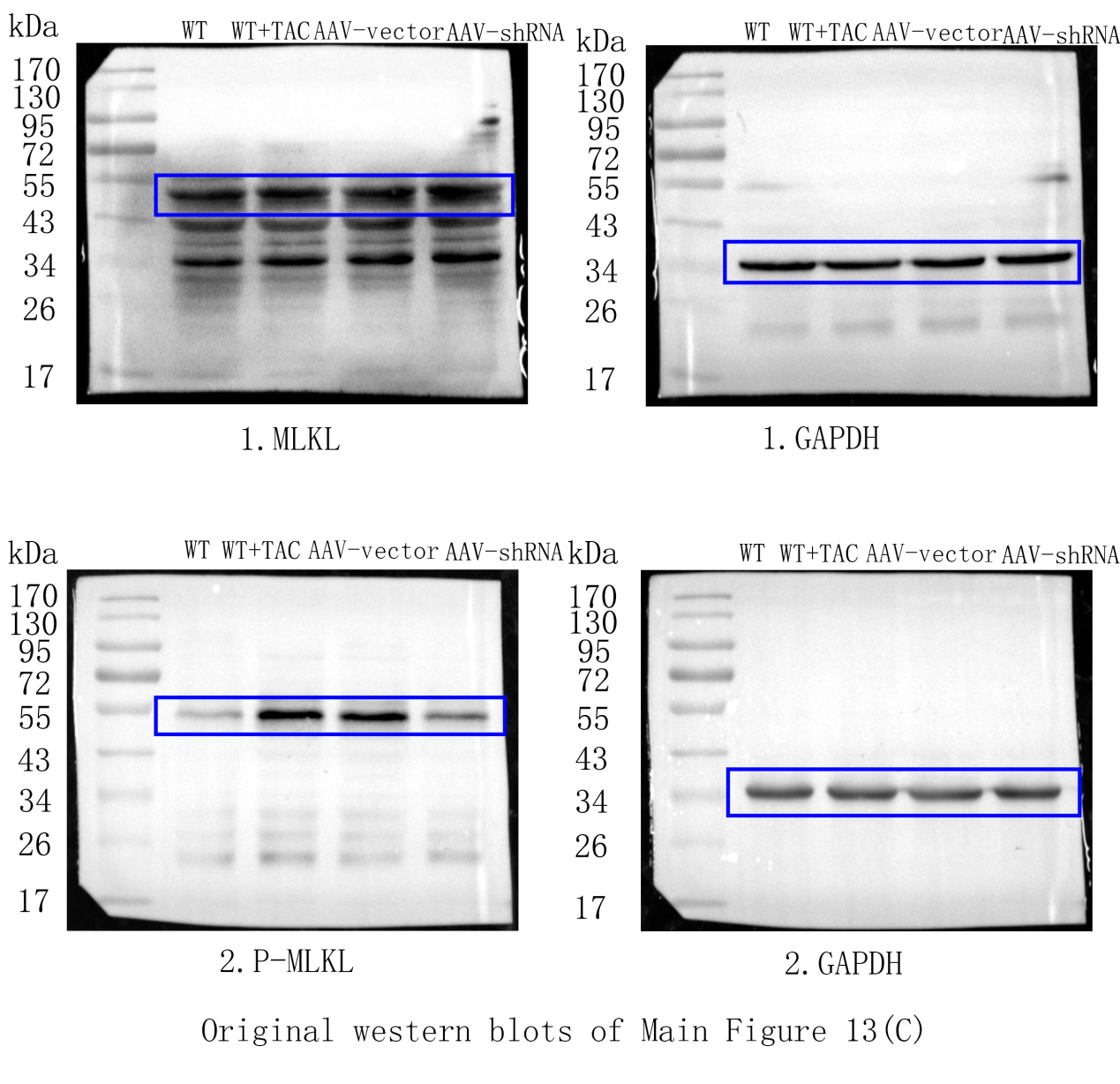

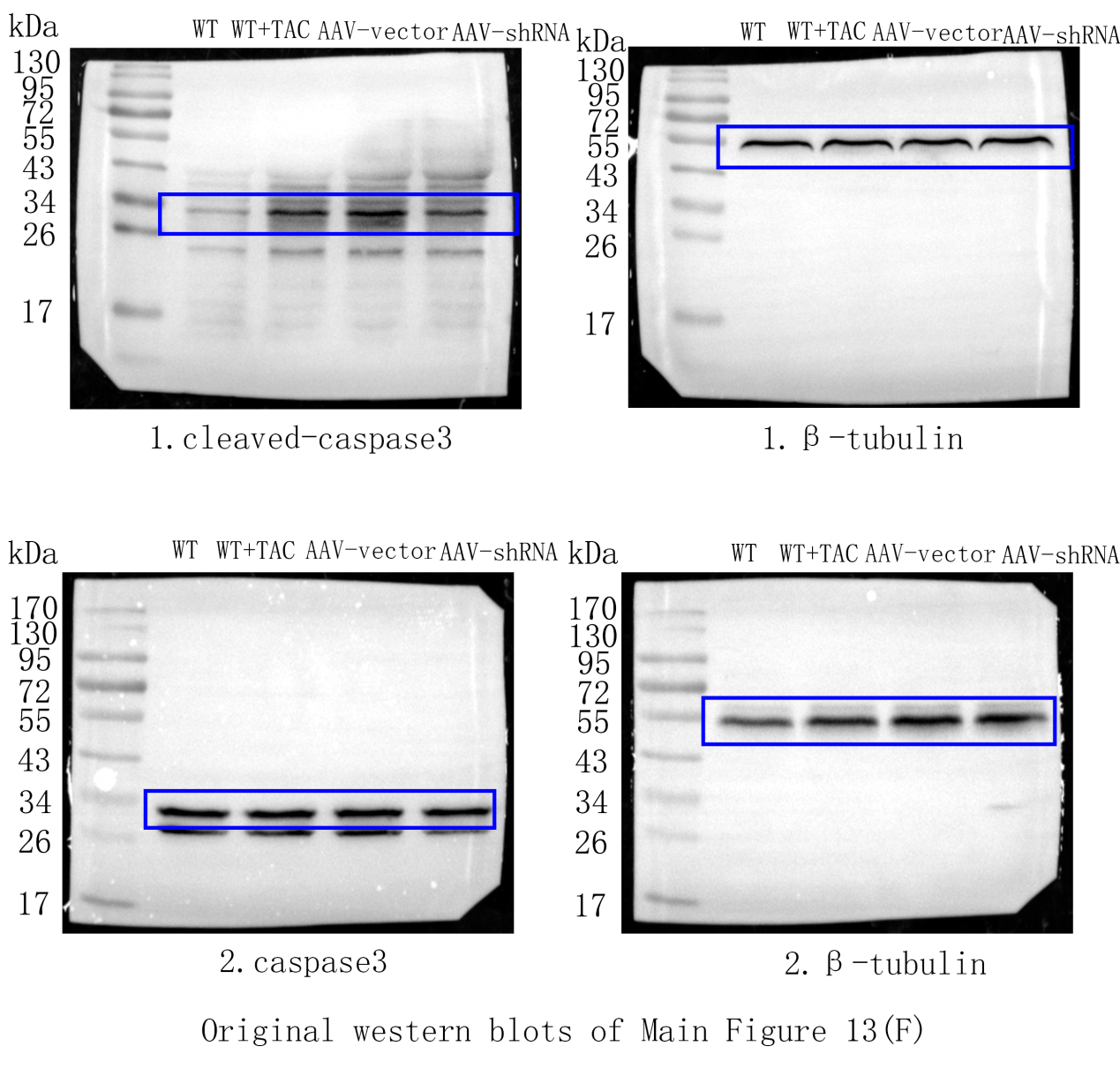

Supplement: Supplementary file 2 [file Data_Sheet_2.DOCX]
